# Tumor Heterogeneity: Mechanisms and Bases for a Reliable Application of Molecular Marker Design

**DOI:** 10.3390/ijms13021951

**Published:** 2012-02-13

**Authors:** Salvador J. Diaz-Cano

**Affiliations:** Department Histopathology, King’s College Hospital and King’s Health Partners, Denmark Hill, London SE5 9RS, UK; E-Mail: sdiaz-cano@nhs.net; Tel.: +44-20-3299-3041; Fax: +44-20-3299-3670

**Keywords:** neoplasm, tumor heterogeneity, topographic compartments, tumor microenvironment, tumor hypoxia, exosome, clonal expansion, cell segregation, tumor progression, metastasis

## Abstract

Tumor heterogeneity is a confusing finding in the assessment of neoplasms, potentially resulting in inaccurate diagnostic, prognostic and predictive tests. This tumor heterogeneity is not always a random and unpredictable phenomenon, whose knowledge helps designing better tests. The biologic reasons for this intratumoral heterogeneity would then be important to understand both the natural history of neoplasms and the selection of test samples for reliable analysis. The main factors contributing to intratumoral heterogeneity inducing gene abnormalities or modifying its expression include: the gradient ischemic level within neoplasms, the action of tumor microenvironment (bidirectional interaction between tumor cells and stroma), mechanisms of intercellular transference of genetic information (exosomes), and differential mechanisms of sequence-independent modifications of genetic material and proteins. The intratumoral heterogeneity is at the origin of tumor progression and it is also the byproduct of the selection process during progression. Any analysis of heterogeneity mechanisms must be integrated within the process of segregation of genetic changes in tumor cells during the clonal expansion and progression of neoplasms. The evaluation of these mechanisms must also consider the redundancy and pleiotropism of molecular pathways, for which appropriate surrogate markers would support the presence or not of heterogeneous genetics and the main mechanisms responsible. This knowledge would constitute a solid scientific background for future therapeutic planning.

## 1. Introduction

Intratumor heterogeneity in human tumors is a widespread phenomenon of critical importance for tumor progression and the response to therapeutic intervention, and it is a key variable to understand tumor natural history and potential response to therapy. It is inherent to neoplasms from early stages and is also the byproduct of tumor progression as genetic abnormalities accumulate. It has normally been assumed that tumor progression is a linear process, with metastasis being a late event, but this model would not match well with the heterogeneity: the invasive capability can be acquired early and result in metastasis from early neoplasms that already show genetic and kinetic features of established malignancies, even for those of low nuclear grade. Human cancers frequently display substantial intra-tumor heterogeneity in virtually all-distinguishable phenotypic features, such as cellular morphology, gene expression, metabolism, motility, and proliferative, immunogenic, angiogenic, and metastatic potential [1–3].

Any current general definition of neoplasm (“cellular disease characterized by abnormal growth regulatory mechanisms”) is descriptive and difficult to apply routinely; working definitions are required. Biologically, neoplasms develop through acquisition of capabilities that involve tumor cell aspects and modified microenvironment interactions, resulting in unrestricted growth due to a stepwise accumulation of cooperative genetic alterations that affect key molecular pathways. The correlation of these molecular aspects with morphological changes is essential for better understanding of essential concepts as early neoplasms/precancerous lesions, progression/blocked differentiation, and intratumor heterogeneity [4,5]. The acquired capabilities include self-maintained replication (cell cycle dysregulation), extended cell survival (cell cycle arrest, apoptosis dysregulation, and replicative lifespan), genetic instability (chromosomal and microsatellite), changes of chromatin, transcription and epigenetics, mobilization of cellular resources, and modified microenvironment interactions (tumor cells, stromal cells, extracellular, endothelium). The acquired capabilities defining neoplasms are the hallmarks of cancer, but they also comprise useful tools to improve diagnosis and prognosis, as well as potential therapeutic targets.

**Figure 1:**
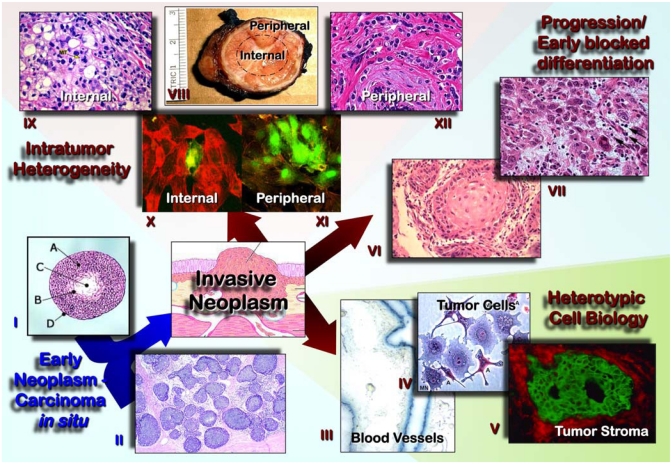
Morphological evaluation of neoplasms and development of oncology. Histopathology, as the gold standard of tumor diagnosis, has set up the bases and criteria for the concept of early neoplasms (I showing encapsulated tumor of epithelial cells, A, with central area of edema, C, and a transitional zone, B; D represent the tumor capsule) and carcinomas *in situ* (II shows a breast lobular carcinoma *in situ*). Histopathology is contributing to a better understanding of the heterotypic cell biology (III to V reveal tumor cells, blood vessels, inflammatory/immune cells, and stroma; tumor cells and stroma are highlighted by direct immunofluorescence for E-cadherin, green, and integrin, red), the biologic progression/dedifferentiation that has been linked to hypoxic conditions and has been frequently reported in recurrent tumors (VI and VII shows well and poorly differentiated squamous cell carcinoma, respectively, in a recurrent neoplasm), and the intratumor heterogeneity and segregation of tumor cells in predominantly expansile internal compartments IX and X) and predominantly invasive peripheral compartments (XI and XII). The expression of hypoxia up-regulated genes is predominantly observed in the internal compartments (green immunofluorescence for HIF-1α in the example).

The introduction of new markers has improved the diagnostic precision, but can potentially result in major changes in prevalence and uncertainties for particular lesions. The current WHO classifications of tumors incorporate new developments based in pathology and genetics, the leading criteria still being morphological: molecular findings complement the histological evaluation without replacing it. The acquired capabilities of neoplasms include tumor cell aspects (self-maintained replication, longer cell survival, genetic instability), and the interaction tumor cell-tissue microenvironment (induction of neoangiogenesis, invasion and metastasis) [6]. The development of clinically detectable tumors requires the accumulation of a number of cooperative genetic alterations, regardless of its order [7]; the evidence available suggest that 5–7 genetic alterations are required for clinically detectable tumors, correlating with morphological progression in some locations. These capabilities are not equally relevant at different stages during tumorigenesis, as highlighted by careful morphological evaluations (Figure 1).

## 2. Tumor Cell Segregation

**Figure 2:**
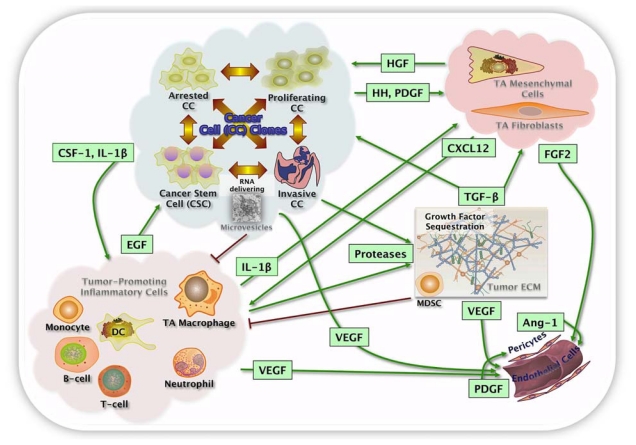
Heterotypic cell biology of tumors and microenvironment. Distinct cell types constitute most solid tumors for both tumor parenchyma and stroma that collectively enable tumor growth and progression. Cancer cells (CC) comprise clones with differential capabilities for kinetics (proliferating and arrested CC), invasiveness (invasive CC) and stemness features (cancer stem cells, CSC). Tumor stroma includes tumor associated mesenchymal cells and fibroblasts, tumor-promoting inflammatory cells, marrow-derived suppressor cells (MDSC) and the vascular component (endothelial cells and pericytes). The multiple stromal cell types create a succession of tumor microenvironments that change as tumors invade normal tissue and thereafter seed and colonize distant tissues. The abundance, histologic organization, and phenotypic characteristics of the stromal cell types, as well as of the extracellular matrix (ECM), evolve during progression, thereby enabling primary, invasive, and then metastatic growth. The assembly and collective contributions of the assorted cell types constituting the tumor microenvironment are orchestrated and maintained by reciprocal heterotypic signaling interactions, of which only a few are illustrated. The signaling depicted within the tumor microenvironment is not static but instead changes during tumor progression as a result of reciprocal signaling interactions between cancer cells and stromal cells that convey the increasingly aggressive phenotypes that underlie growth, dormancy, invasion, and metastatic dissemination. Importantly, the predisposition to spawn metastatic lesions can begin early, being influenced by the differentiation program of the normal cell-of-origin or by initiating oncogenic lesions. Cancer stem cells may be variably involved in some or all of the different stages of primary tumorigenesis and metastasis.

Intratumor heterogeneity comprises both tumor cells and heterotypic components (immune/inflammatory cells, mesenchymal cells, vascular structures, and extracellular matrix (ECM); Figure 2). Intratumor heterogeneity is assumed to occur randomly, but some factors like topography control the segregation of tumor cells within neoplasms [8–10]. The topographic intratumor heterogeneity suggests a differential selection of tumor cells, but can also be expression of either selective clonal evolution or a simple passive byproduct of genetic instability [11]. The differential kinetic profile by topographic compartments has been related with lower cell turnover and apoptosis down-regulation in deep/peripheral compartments, resulting in accumulation of genetic alterations and segregation of tumor cells with differential genetic backgrounds as demonstrated in the adrenal gland, colon and bladder. This process has been linked with mismatch repair protein down-regulation and it is unlikely to be related with hypoxia, which is more pronounced in central compartments. However, the coexistence of genetic alterations supports a key role in tumorigenesis, the topographic heterogeneity resulting from the accumulation of genetic damage. This concept is central and supports multiple sampling to reliably assess the genetic abnormalities of neoplasms. Tumor development can be regarded as a process of cell selection. Indeed, large numbers of cell divisions are required for the emergence of full-blown malignancies and increased genetic instability, presenting plenty of opportunities for the emergence of multiple mutants. This genetic heterogeneity translates into phenotypic heterogeneity, and heritable phenotypes will in turn provide material for selection forces to work on. However, it is likely that a substantial fraction of phenotypic heterogeneity seen in tumors can arise from phenotypic plasticity and differentiation of cancer stem cells (CSC) and is therefore non-heritable.

The heterotypic biology of neoplasms is an essential element to understand tumor growth. The underlying defect (clonal genetic alteration) may reside in stromal and not tumor cells, as reported in juvenile polyposis syndrome and ulcerative colitis hamartomatous polyps [12]. This finding suggests that, at least initially, the stromal cells are the neoplastic cells whose secreting factors drive the epithelial proliferation, and might thus eventually also be responsible for the induction of epithelial malignancy. This bystander role, mutations inducing stromal abnormalities that in turn induce epithelial neoplasia, has been called a *landscaper* effect: the microenvironment surrounding epithelial cells as a major determinant of the disturbed epithelial architecture, differentiation, and proliferation.

### 2.1. Clonal Origin and Expansions. Role in the Natural History of Neoplasms, Tumor Progression, and Intra-Tumor Clonal Diversity

The existence of clonal heterogeneity has been documented for a variety of malignancies, but due to multiple technical challenges, the available data are mostly fragmentary, with the extent of clonal heterogeneity and the dependence of clonal heterogeneity on tumor type, subtype, and disease stage remaining mostly unexplored. It is useful to distinguish cellular genetic heterogeneity (differences at the level of single tumor cells) from clonal genetic heterogeneity (differences that have been amplified by clonal expansion) [13]. Focusing on clonal heterogeneity instead of cellular heterogeneity eliminates some of the “noise” of tumor evolution, as many of the variants detectable at the level of individual cells fail to clonally expand because of their occurrence in a cell that has lost stem cell properties, unfavorable effects on fitness, or simple stochastic reasons. However, “clonal heterogeneity” will not necessarily be completely “noise-free”, as clonal expansion does not necessarily prove the selective value of a mutation.

Neoplasms are not static entities: they start from a genetically normal cell and conclude with billions of malignant cells that have accumulated large numbers of mutations during tumorigenesis, including the emergence of positively selected mutations (“drivers”) and the accumulation of neutral variation (“passengers”) [14,15]. Clonality is a key concept for our current understanding of tumor biology and comprises both clonal origin and expansions, which contribute to both tumor initiation and promotion [16–19]. Clonality tests cannot be interpreted in isolation; they will be meaningless without knowing the effect of a particular marker on cellular kinetics and the interrelationships of that marker with other genetic alterations that are present in a given neoplasm. This dynamic aspect is essential to get robust results and to avoid misinterpretations that might devalue the findings. As with many other issues in tumor biology, it cannot be based on single markers. A complementary approach that takes into consideration the technical limitations is essential to avoid the problems. Several markers have been used to assess tumor clonality [17,18,20], including X-chromosome inactivation, loss of heterozygosity (in particular targeting polymorphic regions of tumor suppressor genes), and mutation analysis.

The value and information provided by clonality markers must be interpreted in the context of the natural history of neoplasms, the scientific methods for test analysis and the test limitations. Neoplastic cells reveal genetic alterations that explain the acquisition of autonomous growth (advantageous cell kinetics) and invasion capacity (local and distant), most of them acquired. This constellation of alterations is most likely related with multiple cooperative genetic abnormalities that explain the biologic and clinical progression [4,5]. In this scenario, we need to consider that the first genetic alteration has not to be necessarily the irreversible abnormality leading to a clinically detectable neoplasm, because genetic alterations can link to apoptosis or may be counterbalanced by other genetic changes resulting in no clinical growth. In inherited cancer syndromes, the first genetic alteration is known, but on its own does not explain clonal growth, the neoplastic lesion displaying additional alterations that correlate with the clinical presentation [17,18]. There are also genetic alterations such as fusion genes described in neoplasms and thought to be an initiating event, also present in inflammatory conditions. In these circumstances, the evaluation will depend on the agreed definition of a given neoplasm [21]. The common finding in all these scenarios is that knowing the first genetic event does not guarantee a clonal growth, unless the additional collaborative alterations support an advantageous cell kinetic resulting in a neoplasm [8,9,19,22–25]. For these reasons a robust clonality test must consider evaluating multiple markers that together can support or refuse a common progenitor for the lesion.

The scientific method to analyze clonality must take into account many unknown variables. Neoplasms progress through a multistep process in which they acquire genetic abnormalities, but the number of genetic alterations, and the sequence of these alterations, is not known [4,5]. It should also be noted that, from a perspective of selection operating in the evolution of tumors, stable, heritable changes in gene expression due to epigenetic alterations are indistinguishable from similar changes caused by alterations in DNA sequences. Silencing of gene expression by hypermethylation of promoter regions is frequently observed in cancers [26,27]; therefore, heritable epigenetic changes should be included in considerations of clonal evolution. In frequent neoplasms, we know the most frequent sequence of genetic abnormalities, but this is not going to be the necessary pathway for all neoplasms in a particular location. Considering all these “uncertainties”, the most sensible approach will be the statistical one by testing the so-called null hypothesis. In clonality analyses, the null hypothesis to test will be if the samples are different, in other words if they come from different progenitors. This hypothesis does not assure that the samples are identical or are derived from the same progenitor. The strength of the analysis will depend on the number of markers tested and the percentage of informative cases for each marker in a normal sample. The higher the values for these two parameters, the more reliable are the results obtained. Testing pathway-independent and dependent markers would be the most sensible approach for clonality assays, including those contributing to the acquired capabilities and processes already identified in neoplasms [4,5]. In that sense, clonality assays must fulfill as much as possible the general requirements for any ideal molecular marker [4,5]. Partial approaches are valid, but they will not provide the same strength.

As with any other technique, clonality assays have limitations. The main limitations would be the heterotypic biology of solid tumors (tumor and nontumor cells coexisting in the neoplasm and the biologic heterogeneity as a byproduct of tumor progression) and the technical aspect to prevent artifacts [4,28,29]. The dynamic nature of neoplasms must be taken into account; there is a continuous selection process of tumor cells that contributes to biologic progression and results in segregation of genetic abnormalities by conditions or topography [10,23–25,30]. Detailed sampling protocols to consider predictable heterogeneity (such as the topographic heterogeneity) and protocols including quality controls for relevant steps during the tests are absolute requirements to have robust results [17,18,28].

#### 2.1.1. Early Neoplasms, Precancerous Lesions and Progression

Different types of “precancerous” or “premalignant” lesions are often used inappropriately; in fact, many of those lesions “are not *pre*-anything”. Four types of lesions based on their fate can be considered: (I) those that progress to more advanced stages, including cancer; (II) those that continue to grow without qualitative change; (III) those persisting with no or minimal growth and no qualitative change; (IV) those that may regress. Alternative pathways of progression from intraepithelial neoplasms must be considered to understand tumor natural history. Tumor heterogeneity and fully established genetic and kinetic features of malignancy in intraepithelial neoplasms and topographic compartments of invasive malignancies are key factors for this process. These two concepts are closely related with tumor initiation, have been developed for epithelial neoplasms and corroborate the concept of multistep tumorigenesis and accumulation of cooperative genetic abnormalities (“gatekeeper” and “caretaker” pathways) [7]. The paradigm is the concept of intraepithelial neoplasm/malignancy, being more difficult to extrapolate the concept to non-epithelial lesions. These lesions would be meaningful when they are present in structures with anatomical boundaries and the cells do not recirculate/migrate in physiologic conditions, regardless of the lesion size. Regarding anatomical considerations, it is the basement membrane, and not the tumor capsule, the limiting structure.

Although some genetic alterations have been proposed as neoplasm-specific, the presence of a single genetic alteration cannot be considered diagnostic of malignancy even for early stages. These problems preclude establishing reliable diagnoses of follicular carcinomas *in situ* for encapsulated neoplasms carrying PAX8/PPARγ fusion genes, or lymphomas *in situ*, even for lesions that initially carry molecular changes reported in malignancy. The opposite situation is equally important: Histologically confirmed intraepithelial lesions are considered precursors, but they can accumulate genetic alterations and show kinetic features of malignancies, as reported in MEN 2A [25,31].

Although the existence of intra-tumor phenotypic heterogeneity has been recognized from the early days of experimental cancer research, the relative contributions of heritable and non-heritable mechanisms are still not clear, and yet the nature of tumor heterogeneity can have profound implications both for tumor development and therapeutic outcomes. Tumor evolution has often been depicted as successions of initiations and promotions of mutated cells in clonal expansion rounds, where every new round is driven by the acquisition of additional kinetically-advantageous mutational events (selection process) [19]. This sequential process of stochastic acquisition of key mutations drives tumor progression, as a result of proliferation and increased genomic instability that produce progressive selection [9,10,30,32]. Only minority of random mutations are selectively advantageous, while a large fraction of mutations will be discarded by selection. Furthermore, many neutral or even mildly disadvantageous mutations can be retained in the population or even undergo some expansion due to genetic drift. Moreover, the long-term evolutionary success of mutations providing a positive selective advantage is not granted. As a consequence, some of the mutations that are selectively advantageous at certain stages of tumor progression and can trigger substantial clonal expansion may lead to evolutionary dead ends and, therefore, may not be present in a fully malignant tumor. The complexity of tumor evolution is further influenced by the ongoing alterations of tumor microenvironment associated with tumor progression, [33] which are likely to alter the selective pressures experienced by tumor cells. Therefore, at the microscopic level, tumor evolution is likely to be non-linear, and substantial genetic heterogeneity is expected in tumor cell populations [10,23,30,31,34].

### 2.2. Cancer Stem Cells and Plasticity

It is important to stress that the concepts of clonal evolution and CSCs are complementary rather than mutually exclusive. Tumor progression is contingent on acquiring specific heritable mutations in oncogenes, tumor suppressor genes and genome maintenance genes. These genetic alterations must target cells with the unique ability to both limitless self-renewal and multipotential differentiation to explain tumor growth and progression. As cells with these unique features, the so-called “CSCs”, represent only a minor fraction of tumor cells, the majority of tumor cells are considered to be products of abnormal differentiation of CSCs, and, although some might be capable of limited proliferation, they represent evolutionary “dead ends.” From an evolutionary perspective, limitation of self-replicating capacity to a fraction of tumor cells means that the effective population size is restricted to this stem-like compartment, rather than encompassing a bulk of tumor cells. The implication is that phenotypic and genetic heterogeneity, associated with tumor stem cell differentiation, are irrelevant for tumor progression (as long as they do not affect the tumor stem cell subpopulation or lead to stem cell conversion), as selection can only work on the heritable phenotypes of CSCs (Figure 2).

In recent years, evidence accumulated suggesting that a small tumor cell subpopulation in the primary tumor mass might be responsible for tumor initiation, growth, maintenance and spreading. These cells, termed CSCs or cancer initiating cells (CSC/CIC), represent a population with stem cell-like properties, in particular long-term survival, high self-renewal and seeding capacities [35]. While the cellular origin of CSC/CIC and the associated molecular pathways are still a matter of discussion, the existence of a small tumor cell subpopulation capable of initiating and maintaining tumor growth and initiating metastasis is being increasingly documented and accepted [36,37]. Furthermore, evidence indicates that CSC/CICs are more resistant to classical therapeutic approaches (*i.e.*, chemotherapy and radiotherapy) compared to the bulk of the tumor cell mass. The mechanism of resistance remains largely elusive and might include the increased expression of multi-drug resistance-type of membrane transporters, a protective effect of the microenvironment or especial resistance to apoptosis. The existence of morphologically defined subsets of cancer cells, which are enriched with the ability to form tumors in xenograft models, has been demonstrated for many hematopoietic and solid malignancies. Initial evidence for the existence of CSC/CIC came from acute myeloid leukemia rare leukemic (stem) cells with a CD34^+^/CD38^−^ phenotype, and subsequently demonstrated in solid tumors, but with different surface phenotype [38,39]. In all cases, CSC/CIC is distinguished from the “somatic” tumor cell population, by their capacity to efficiently generate new tumors when implanted at low number in mice. Conversely, “somatic” cancer cells are not able to initiate tumor growth, even in high number, in the same *in vivo* preclinical models. A major challenge to the CSC concept originates from the observation that its definition is based on experimental evidence (e.g., tumor initiation at limited dilution) that is highly subject to how the assay was performed. For example, in experimentally induced or spontaneous cancers, the majority of cancer cells are capable of initiating tumors in either syngenic or xenograft models [40], and thus can be considered CSCs. When most of the cells in a tumor are CSCs, singling them out becomes meaningless. It has also been noted that numerical considerations frequently reveal inconsistencies with data interpretation in experiments transplanting sorted cell populations [41]. In addition, comparisons of the genetic composition of breast cancer CD44^+^CD24^−^ cells, which are presumed to be “CSCs”, *versus* CD44^−^CD24^+^ cells, which are considered to be “non-stem cells”, have revealed that in some cases these subpopulations are genetically divergent [42,43], which is inconsistent with a model of simple differentiation.

#### 2.2.1. Phenotypic Plasticity

Tumor cell plasticity explains the differential ability of specific subsets of tumor cells to initiate tumors in experimental models. This hypothesis proposes that the majority of tumor cells reveals stem cell features with varying degrees of “stemness”, where the “stemness” is influenced by microenvironmental cues and some stochastic cell-autonomous mechanisms [44]. Observation of substantial phenotypic and functional heterogeneity within normal stem/progenitor cell pools has led to organizational models of stem cell compartments that incorporate self-organization, flexibility, and plasticity of stem cell properties [45–47]. Importantly, mathematical models that involve plasticity of the stem cell phenotype provide a much better match to experimental data on stem cell dynamics than does the concept of rigid differentiation hierarchy [14,41,48]. Concepts of CSCs and phenotypic plasticity are not mutually exclusive. Even if the majority of tumor cells in some (or many) cancers are incapable of sustained proliferation and, therefore, can be described as non-stem cells, the stem cell compartment can still be phenotypically diverse and plastic. Regardless of the outcome of the CSC debate, it is likely that non-heritable mechanisms are responsible for a large fraction of intra-tumor heterogeneity of cellular phenotypes.

Genotypes specify a range of phenotypic manifestations within a norm of reaction. This concept would explain the tumor cell ability of altering their phenotype in response to microenvironmental cues and, as a result of this tumor cell-microenvironment interaction, the tumor behavior and progression are shaped. However, the tumor microenvironment is not completely homogeneous: different regions of a tumor can have different densities of blood and lymphatic vasculature, different numbers and types of infiltrating normal cells, and different composition of ECM. Therefore, tumor cells within a given tumor are expected to experience a range of microenvironmental cues, which would translate into a range of phenotypic manifestations. In addition, cells can show heterogeneity of features even within apparently homogeneous environments. Such heterogeneity arises from noise in gene expression and existence of meta-stable configuration of intracellular networks, and it is a very basic feature of all living cells. Normal and cancer cell lines display substantial heterogeneity in timing of apoptotic response to TRAIL ligand. This heterogeneity does not depend on genetic or epigenetic mechanisms but is instead caused by apparently noise-driven differences in levels of protein expression [49]. Plasticity of tumor cell phenotype is not limited to apoptotic response. For example, genetically homogeneous tumor cell lines display morphological heterogeneity, as mixtures of immotile, rounded cells and motile, fibroblast-like ones can be found both *in vitro* and *in vivo*. In this case, the phenotypic differences result from different, mutually exclusive, and inter-convertible activation of Rac and Rho GTPases [50].

### 2.3. Interactions of Distinct Tumor Clones. Clonal Evolution and Progression

Two main mechanisms explain the impact of clonal heterogeneity on tumor evolution, modulating both progression and therapeutic escape: (a) Clonal diversity provides a more diverse genetic spectrum on which selection can take place; (b) The co-existence of genetically distinct clones within a tumor gives a network of biological interactions among distinct clones. As a consequence, the behavior of a tumor composed of distinct clones might be different from that of a monoclonal tumor or the behavior of the sum of the individual clones [15,33].

#### 2.3.1. Tumor Evolution as Byproduct of Clonal Heterogeneity

Tumorigenesis is a dynamic selective process driven by genetic changes (mutations in broader terms) and abnormal gene expression [51]. In this context, higher genetic complexity is expected to provide more options for selection and a faster pace of evolution in heterogeneous neoplasms. As tumors grow, the cellular stress derived from replication results in mutations and expansion of sub-clones from the branching of the original precursor (monoclonal population). This selection results in an increased genetic complexity generated by branching from a tumor composed of multiple distinct clones. Thus, clonally heterogeneous tumors can generate a larger variety of genetic variants to be tested by selection, which provide a wider adaptive landscape, increasing the probability of clones reaching fitness for challenges from several microenvironments (Figure 2).

The clonal composition of tumors can be especially important in determining responses to dramatic changes in the environment, such as changes induced by anti-cancer therapy. In this case, the pre-existence of resistant clones within a tumor can make the difference between tumor extinction (treatment success) and tumor evolutionary adaptation (treatment failure). A vivid illustration of the importance of clonal heterogeneity in therapeutic resistance can be found in malignancies such as chronic myelogenous leukemia [52], gastrointestinal stromal tumors (predominantly driven by different activating mutations in KIT) [53], tumors associated with inactivating mutations in BRCA1 and BRCA2 (deficient in homologous recombination-mediated DNA repair resulting in genomic instability and highly sensitive to platinum compounds and poly ADP ribose polymerase inhibitors) [54], just to give some examples. For many tumors with genetic mechanisms of resistance pre-existence of resistant cell types prior to treatment has yet to be demonstrated. Moreover, the generality of the mutational mechanisms of acquiring therapeutic resistance remains an open question [55]. Nonetheless, intra-tumor heterogeneity is likely to represent a strong challenge to therapeutic success, as larger genetic diversity within a tumor would be expected to increase the probability of the pre-existence of resistant cell types that could be selected by treatment and ultimately result in relapse of resistant tumors. Notably, in addition to the scenario of cancer therapy, selection of mutant variants rather than mutagenesis was proposed to be the key mechanism responsible for the carcinogenic action of a wide range of growth-limiting carcinogens [55–57].

#### 2.3.2. Biological Interactions among Distinct Tumor Clones

**Figure 3:**
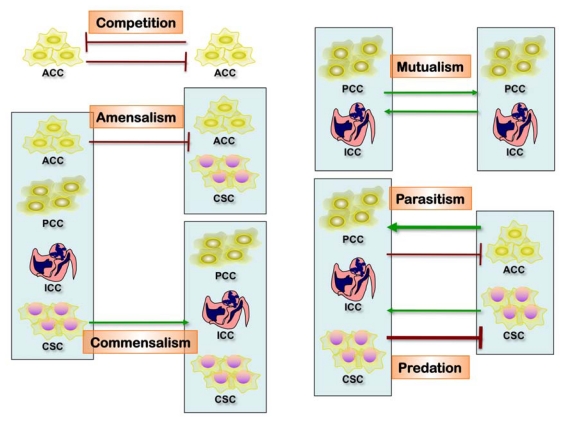
Interaction between tumor cell clones. This process includes competition, amensalism, commensalism, mutualism, parasitism and predation; the outcome is a higher level of complexity of tumor heterogeneity at the cellular level.

Cancer morphologic and genetic heterogeneity is the expression of multistep tumorigenesis that leads to subclonal tumor cell populations with heritable traits (including primary, circulating and metastatic cells) and a microenvironment of ECM, fibroblasts, inflammatory cells and blood vessels. In addition, the differentiation hierarchy within tumors complicates the network, along with the importance of tumor microenvironment, as it is increasingly clear that understanding alterations within tumor cells is only part of the picture, and we need to understand interactions between tumors and their microenvironment to account for multiple aspects of tumor progression and therapeutic resilience [33,58,59]. A major implication is the co-existence of phenotypically distinct clonal populations of tumor cells should inevitably lead to the formation of a network of biological interactions, which could be either direct or mediated by the tumor microenvironment. Some of the key interactions that are likely to exist between distinct tumor clones are summarized below (Figure 3) [60].

*Competition* is likely to be the strongest and most important biological interaction between tumor cells and it is one of the mechanisms involved in the tumor response to irradiation [61]. Mechanisms to limit the competitive outgrowth of mutant cells include intrinsic tumor-suppressive mechanisms, wherein activation of strong oncogenes triggers activation of tumor-suppressive networks, resulting in senescence or death of mutated cells [62]; or oncogenic mutations that can trigger stronger proliferation without engaging intracellular tumor suppressors resulting in loss of stemness (extrinsic tumor suppressor mechanisms) [63]. Moreover, the spatial organization of normal tissues can limit the role of competition between genetically distinct cells even further, confining stem cells to small pools [64,65]. However, carcinogenic and growth-limiting conditions can substantially modify the fitness landscape, allowing for the competitive outgrowth of oncogenically mutated cells, thereby initiating malignant evolution [66,67]. Tumor progression is associated with further loss of tumor-suppressive mechanisms and disintegration of normal tissue morphology; thus, tumors start to resemble ecological systems rather than integrated tissues, and competition becomes the strongest biological interaction. The role of competition is further strengthened by the limited nature of resources: while, under tissue culture conditions, tumor cells are capable of limitless exponential growth, clonal expansion within tumors is severely constrained by the limited availability of oxygen, nutrients, growth factors, and space (habitable niches). Limited resources intensify competitive interactions both within and between interacting species (cancer subclones). In large, spatially homogeneous populations, competitive interaction results in the fixation of a clone with the highest fitness value [60,64,65]. However, as discussed above, fixation can be inhibited by spatial organization, which limits competition to within clones. In addition, the existence of regions with different selective pressures can mediate the co-existence of clonally distinct populations. Finally, a stable co-existence of multiple clones is also possible when fitness is density-dependent [68].

*Amensalism* is an interaction in which one interacting party is inhibited by the other without being affected itself. Under the competitive context, this interaction is also referred to as “interference competition”. As tumors grow under conditions of limited resources, amensalistic interactions can provide a competitive advantage to a clone that can inhibit other clones while being (at least relatively) resistant. An example of this type of interaction between genetically distinct human cells can be found in Bcr-Abl-driven leukemias [69]. The existence of this type of interaction has also been documented among distinct tumor clonal populations, both in cell culture and *in vivo.* The concept of amensalism can be extended to interactions between primary and metastatic tumors, as many human and experimental tumors can suppress metastatic outgrowth by inhibiting angiogenesis or inducing dormancy of single disseminated tumor cells through uncharacterized mechanisms [70]. Thus, amensalism can work as a weapon in competitive “warfare” between distinct clonal populations, which can lead to the stable co-existence of distinct clones in tumors.

*Antagonism* is an interaction in which one interacting party can capture biomass from the other one. This interaction is widespread in natural ecosystems, and there are clear parallels between organism–tumor and host–parasite interactions. However, antagonism is unlikely to be relevant for the interactions between distinct tumor clones.

*Commensalism* is a positive interaction in which one interacting party benefits the other without itself being affected. The tumor cell-stromal cell interaction in itself is a form of commensalism, because it has been demonstrated that these non-malignant cells support and even enable tumor growth [71]. An example of this type of interaction can be found in normal tissues: mammary epithelial cells from estrogen receptor null mice (ERα−/−) fail to grow and do not develop branching structures upon transplantation into cleared fat pads. However, when mixed with wild-type epithelial progenitors, ERα−/− cells do proliferate and contribute to different lineages and different parts of the mammary gland [72]. In addition, nearby cells can protect each other from a set of host defenses that neither could survive alone. Cooperation can evolve as by-product mutualism among genetically diverse tumor cells [71]. A recent publication from the Weinberg laboratory demonstrated the relevance of this interaction in a human xenograft model, where an “instigator” tumor cell line augmented the proliferation and metastasis of genetically distinct “indolent” tumors that were incapable of forming macroscopic outgrowths on their own [73,74]. In this case, the “instigation” was mediated by systemic effects, which could at least partially be attributed to the secretion of SPP1 (osteopontin) by the “instigator” cells [74].

Although not experimentally validated, this interaction can account for the co-existence of clonal variants that differ in their angiogenic potential. If the fitness benefit of angiogenic factor production is higher than the associated fitness cost, an angiogenic clone can undergo competitive expansion and reach stable equilibrium with non-angiogenic “free rider” clones. Interestingly, mathematical modeling shows that, under certain physiologic conditions, “free riders” capable of faster proliferation can out-compete the angiogenic clone, leading to the collapse of the tumor [68]. This prediction can potentially explain spontaneous regression of neuroblastomas accompanied by massive necrosis [75].

*Mutualism* (cooperation) is a positive interaction in which both interacting parties can benefit from each other. Positive interactions between species have been widely documented in natural ecosystems. Mathematical modeling suggests that mutualistic interactions can lead to the co-existence of distinct species even under competitive contexts [71,76]. It has been suggested that mutualistic interactions between distinct tumor clones can play an important role in tumor evolution by maintaining the survival and proliferation of tumor cells until one of the clones achieves a “full deck” of malignant mutations required for clonal dominance and full-blown malignancy [71,77]. To the best of our knowledge, however, such interactions between distinct clonal tumor populations have not been experimentally documented.

Of note, most of the biological interactions are not mutually exclusive, and the net outcome for the interacting species will depend on the net sum of the different interactions (Figure 3). This net result can lead to both augmentation and retardation of overall tumor growth and progression. Elucidation of biological interactions between populations of tumor cells might be a very formidable task; however, it can deepen our understanding of tumor biology and uncover new therapeutic targets.

## 3. Tumor Components

Normal cells survive and grow within defined environmental niches and are subjected to microenvironmental control. Outside of their specific niche, the tissue environment is hostile to normal cells. Since they lack necessary cell autonomous survival signals, normal cells will not survive an inappropriate microenvironment [78]. Detachment-induced cell death (anoikis) has been proposed as the mechanism preventing normal cells from leaving their original environment and seeding at inappropriate locations [4]. In order to evade local tissue control and avoid anoikis during tumor development and progression, malignant cells start interacting with the surrounding ECM [79]. A bidirectional relationship is initiated between tumor cells and its surrounding stroma as a first step to invasive growth on metastatic spreading. Stromal changes sustaining tumor progression include modifications of the ECM composition, activation of fibroblasts, myoepithelial cells, and the recruitment of pericytes or smooth muscle cells and immune and inflammatory cells [80].

### 3.1. Cellular Interactions and Microenvironment

Human tumors arise from single cells that have accumulated the necessary number and types of heritable alterations. Each such cell leads to dysregulated growth and eventually the formation of a tumor. Despite their monoclonal origin, at the time of diagnosis most tumors show a striking amount of intratumor heterogeneity in all measurable phenotypes; the evolutionary dynamics of heterogeneity arising during exponential expansion of a tumor cell population, in which heritable alterations confer random fitness changes to cells [48]. Classical multistage modeling of tumorigenesis evolves through the processes of local proliferative lesions (tumor initiation and promotion or selection), and acquisition of invasion-metastatic potential (tumor progression). Broadly speaking, tumor promotion consists of the selective (clonal) expansion of altered cells to form focal lesions [16,81]. Within this definition, the process of promotion is mainly a quantitative phenomenon (many cells arising from a single cell), while no qualitative changes are necessarily implied. However, these latter properties are lost during tumor progression, which is typically characterized by increasing levels of tumor cell heterogeneity. This implies that qualitative changes are now dominant [18,19], generating distinct cellular sub-clones with different phenotypes. Such a background represents the landscape for the full deployment of tumor progression.

These two distinctive processes, the mainly *quantitative* process of tumor promotion and the intrinsically *qualitative* process of tumor progression, are driven by two distinct microenvironments: the tissue and the tumor microenvironments [56,57,82]. The tissue microenvironment specifically refers to the local environment surrounding altered cells during their selective clonal expansion to form focal proliferative lesions. Conversely, the tumor microenvironment describes the unique biological milieu that emerges inside focal proliferative lesions as a consequence of their altered growth pattern [56,57,82]. Such new biological niche is characterized by a tissue architecture which is not developmentally programmed and is bound to pose major challenges for cell survival, due to altered/inadequate supply of oxygen and nutrients. This in turn can lead to biochemical and metabolic alterations that can profoundly impact on the fate of the cell populations inside focal lesions [83].

The available evidence is consistent with the hypothesis that focal lesions result from the clonal expansion of altered cells; if so, how does a focal lesion develop from those rare altered cells? Theoretically, at least two different (and not mutually exclusive) possibilities should be considered:

- The altered/initiated cell is already endowed with some degree of inherent growth autonomy and starts to replicate unchecked, forming a focal lesion and then, after a number of further steps, a full blown cancer [84]. The analysis of several multistage models of cancer induction has led to the conclusion that initiation per se does not result in any significant growth of pre-neoplastic and/or neoplastic lesions, and the appearance of the latter is heavily dependent on the presence of a promoting/selective environment [85]. Furthermore, transplantation experiments have convincingly demonstrated that different types of altered cells do not display any evidence of growth autonomy when transferred in a normal tissue environment of young animals *in vivo* [67]. By analogy, altered, putative initiated cells can be found in the skin of several healthy human subjects, suggesting that their presence per se is not necessarily associated with selective clonal growth, and additional (promoting/selective) influences must be enforced when the latter does occur [86]. Also epidemiologic evidence on smoking and lung cancer suggests that clonal expansion of cells is much more relevant than early mutations [87].
- (II) The other possibility is that the single altered cell does not express any significant degree of growth autonomy and is still under the control of normal homeostatic mechanisms; if this is the case, its selective clonal growth must be linked to the dynamics of cell turnover typical of the tissue where it resides. Thus, specific alterations of these dynamics could translate into a promoting effect for any putative altered cells present in that tissue. It is self-apparent that, within this perspective, the tissue microenvironment surrounding rare initiated/altered cells is given a central role in their selective emergence as focal proliferative lesions. In this context the neoplastic development is a biologic process that is not directly caused by the inciting agent acting on a passive target, but results from the interaction of living structures (cells, tissues and organs) with those agents [88,89].

A direct link was established between growth inhibitory effects and the emergence of phenotypically resistant cell populations during carcinogenesis. Rare initiated cells could withstand certain types of growth suppression imposed on surrounding normal cells, thereby acquiring a proliferative advantage under appropriate “selective” conditions. Furthermore, the growth of such resistant cells to form focal proliferative lesions could be induced by physiological homeostatic stimuli, as it occurs during tissue regeneration and/or turnover, and these rare clones could emerge *because* the bulk of surrounding normal cells were unable to respond to those stimuli [90]. Incidentally, the work of Farber was the first to provide evidence that tumor promotion could be interpreted (and in fact defined) as a process of cell selection, thereby introducing a Darwinian perspective in the analysis of carcinogenesis [91]. Thus, early phases of cancer development, far from being exclusively cell-autonomous, appeared to be heavily dependent on environmental influences and in fact could be interpreted as adaptive reactions to altered conditions in the surrounding tissue [88]. Specifically, if the growth potential of normal cells in a given tissue was severely impaired, this could translate into a driving force for any altered/initiated cells to expand and compensate for the (relative) impairment of their surrounding counterparts. As a consequence, cancer development is a process to be considered and analyzed at tissue/organ level, not just at single cell level, and a role for the tissue microenvironment in this process is clearly defined [12,88,92].

#### 3.1.1. Promoting Potential of a Growth-Constrained Tissue Microenvironment

As the large majority of carcinogens can both (I) exert growth-suppression in the target tissue, possibly as a consequence of the inflicted DNA-damage [93], and (II) induce rare altered/initiated cells with a resistant phenotype [94], a clear outcome of such combined effects includes the possibility of a selective expansion of the resistant cell population [91,93]. Given that random mutations are more likely to damage the function of the genome rather than to improve it [91], the same genotoxic agent can both initiate the carcinogenic process in a given tissue and exert a promoting/selective effect on rare initiated/altered cells by limiting the proliferative potential and/or impose other cytotoxic effects in the bulk of the surrounding cells in that tissue [95].

A direct testing of the hypothesis that a growth-constrained microenvironment can represent a powerful driving force during tumor promotion is provided by cell transplantation experiments. Under growth-constraint endogenous conditions, transplanted altered cells could selectively expand in the recipient liver, forming hepatocyte nodules and eventually progressing to hepatocellular carcinoma. Importantly, no nodular growth was observed when a similar preparation of hepatocytes was injected into normal, untreated recipients [67], suggesting that nodular cells had no inherent growth autonomy: the selective growth of altered cells in this system occurs under the influence of homeostatic mechanisms which are similar to, and possibly coincide with those controlling normal cell turnover [96].

One relevant situation to be considered in this context is *aging*. In fact, aging represents the major risk factor for neoplastic disease (albeit it is not an avoidable risk) [97]; moreover, it is characterized, if not defined, by a generalized decrease in the functional proficiency of several organs and tissues, including a decline in their proliferative potential [98]. It has been shown that the microenvironment of the aged liver is able to support the growth of a transplanted epithelial cell line, while that of the young recipient is not [99], being able of stimulating the clonal expansion of transplanted normal hepatocytes [100]. This phenomenon does suggest that the liver microenvironment associated with aging is also clonogenic, and could therefore foster the emergence of altered cell populations. It appears reasonable to propose that such clonogenic potential might be linked, at least in part, to the growth-constrained environment associated with aging [100]. It is likely that a role in such age-associated clonogenic effect is also played by stromal fibroblasts. Senescent fibroblasts can in fact stimulate early growth of both grafted normal and tumor epithelial cells, suggesting that they can mediate, at least in part, the effect of aging on the parenchymal component of various tissues [101,102]. However, this effect can translate into selective growth of rare altered cells if the majority of surrounding counterparts are relatively impaired in their proliferative capacity, as it occurs during aging [103].

From a more general standing point, *chronic tissue injury and inflammation* lead to both impaired function and an increased risk of neoplastic disease in the target organ [104,105]. It is reasonable to consider that a common factor in all these instances could be the progressive exhaustion of the functional and/or proliferative capacity of parenchymal cells, paving the way to the selection of rare variant cells with an altered phenotype [106]. Consistent with this interpretation is the finding of diffuse or focal parenchymal atrophy concomitant with proliferative lesions of putative clonal origin [107–111]. The “proliferative inflammatory atrophy” of the prostate has been recognized as a risk factor for prostate carcinogenesis and it is characterized by clusters of proliferating prostatic cells arising in areas of atrophic epithelium [108], suggesting their possible regenerative significance [109].

In this context, defects in DNA repair pathways (a known cancer risk) result in increased probability of critical genetic alterations occurring in rare cells, and this will lead to the emergence of the neoplastic phenotype. However, such interpretation largely overlooks the consequences that the defective DNA repair might have on the bulk of the tissue, and their possible contribution to the increased carcinogenic risk that is seen in these conditions. Defects in DNA repair pathways can be associated with accumulation of widespread DNA damage [112]. It is reasonable to assume that such randomly inflicted damage will generally impair genome function [112], rather than improve it; in fact, accelerated aging has also been associated with altered DNA repair capacity [113]. Thus, the above considerations suggest that carcinogenesis related to defective DNA repair is also interpretable as the end result of at least two main biological components: (I) induction of rare altered cells; and (II) selection of such cells within a functionally impaired tissue environment.

#### 3.1.2. Molecular Analysis of Selectogenic Microenvironments

Given that altered cells can be selected in a tissue microenvironment which is otherwise growth-inhibitory to surrounding counterparts, a relevant question pertains to the biochemical and molecular basis of such phenotypic resistance. Blagosklonny has proposed the existence of two broad types of resistance [55]: (I) Non-oncogenic resistance relates to changes in drug metabolism and/or uptake, such that the rare altered cell is able to withstand toxicity compared to the rest of the population in that tissue. Such phenotypic resistance would still translate in the clonal growth of that rare cell, but no increased risk of neoplastic disease would be implied [55]. (II) The oncogenic resistance is linked to the inability of the cell to sense or repair DNA damage and/or to activate effector mechanisms leading to cell cycle arrest and/or cell death. As a result, the affected cell is susceptible to acquire a “mutator phenotype”, *i.e.*, the tendency to undergo a cascade of further mutations [4,8,91,114].

The mutator phenotype has been linked with a defect in mismatch repair (MMR) genes, so that a cascade of mutations occurs in cancer-related genes. To justify the onset of a mutator phenotype in “sporadic cancers” (which are in fact the vast majority) we have to revisit some theories of carcinogenesis and their evidence base [10,32]. In sporadic cancers the origin of the mutator phenotype has been attributed to chance, or to mutagens that selectively affect specific genes similar to MMR genes, or to a combination of the two. However, MMR is clearly mutated only in a minority of cases: For example, colon cancers characterized by the presence of microsatellites (MIN) are a small minority compared to cancers characterized by chromosome instability (CIN), whose onset has not yet been attributed to the failure of any specific gene repair such as MMR [8,114]. To explain the most common type of lesions that are found in non-hereditary cancers, chromosome aberrations and CIN, we have to explain how the mutator phenotype originates. In addition, a key concept that has also emerged recently is that mutations, or instability, are irrelevant if there is not a *microenvironmental change* that selects the cells carrying such mutations. Therefore, we will first discuss some examples of such a “selectogenic” microenvironment.

#### 3.1.3. Stress and the Mutator Phenotype

Cell replication is the main source of cellular stress. On one hand, continuous proliferation results in telomere attrition and reduced stability of chromosome ends, which activate the cycle of chromosomal fusion-bridge-breakage and higher incidence of translocations such as expression of chromosomal instability (CIN). On the other hand, nucleotide mismatches are introduced by DNA polymerase and will accumulate in DNA regions with repetitive sequences, such as microsatellites; this is the basic reason of microsatellite instability (MSI), a finding more frequently detected in tissues with higher proliferation. CIN and MSI have been described as two alternative pathways to cancer [4,8]. CIN is generally defined as the ability of a cell to gain and lose chromosomes and is a feature of many types of cancer. Conversely, microsatellite instability is related to a defect in the DNA mismatch repair machinery (MSI cancers).

The net result of CIN is the deregulation of chromosome number (aneuploidy) and an enhanced rate of loss of heterozygosity, which is an important mechanism of inactivation of tumor suppressor genes. Cytogenetic studies of bladder, lung and colon tumors have shown that karyotype complexity, cell ploidy, and the number of structural changes found are closely associated with tumor grade and stage. It has been suggested that different environmental carcinogens can induce specific forms of genetic instability [115]. The available data demonstrate that exposure to specific carcinogens can indeed select for tumor cells with distinct forms of genetic instability and *vice versa*. These data offer potential clues to one of the remaining unsolved problems in cancer research, the relationship between environmental factors and the genetic abnormalities that effect tumorigenesis.

#### 3.1.4. Chronic Inflammation, Myeloid-Derived Cells in Tissue and Tumor Microenvironment

Chronic inflammation promotes tumor onset and development through nonimmune and immune mechanisms. The nonimmune mechanisms include the following: (I) the production of reactive oxygen species (ROS) such as peroxynitrites, which cause DNA mutations that contribute to genetic instability and the proliferation of malignant cells [116]; (II) the production of proangiogenic factors such as vascular endothelial growth factor (VEGF), which promote tumor neovascularization [117]; and (III) the production of matrix metalloproteases, which facilitate invasion and metastasis [118]. The predominant immune mechanism is the perturbation of myelopoiesis and hemopoiesis, which causes a deficiency in Ag-presenting dendritic cells (DC) and dysfunctional cell-mediated antitumor immunity [119]. A major culprit in this latter deficiency is the production of myeloid-derived suppressor cells (MDSC), an immature population of myeloid cells that is present in most cancer patients and mice with transplanted or spontaneous tumors. Because MDSC inhibit both innate and adaptive immunity, they are likely to subvert immune surveillance and prevent an individual’s immune system from eliminating newly transformed cells. In individuals with established cancer, they are likely to be a major factor in preventing the efficacy of immunotherapies, such as cancer vaccines, that require an immunocompetent host [120,121]. MDSC cause immune suppression in most cancer patients, where they are an impediment to all immunotherapies that require an active immune response by the host. They may also facilitate the transformation of premalignant cells and promote tumor growth and metastasis by suppressing innate and adaptive immune surveillance that would otherwise eliminate abnormal cells. The induction of MDSC by proinflammatory factors identifies the immune system as another contributing mechanism by which chronic inflammation contributes to the onset and progression of cancer.

In the tumor microenvironment, tumor associated macrophages (TAMs) constitute the majority of tumor-infiltrating leukocytes. MDSC have been identified in most patients and experimental mice with tumors based on their ability to suppress T cell activation. The nuclear morphology and content of immunosuppressive substances have also been used to characterize mouse MDSC. Two distinctive TAM sub-populations have been defined. Classical, or M1, macrophages are characterized by the expression of high amounts of iNOS and tumor necrosis factor-α (TNF-α), whereas alternatively activated M2 macrophages typically produce ARG1 and IL-10 [122,123]. At the tumor site in wild-type mice, TAMs are predominantly M2-like macrophages, which are the cells primarily responsible for suppressing T cell-mediated antitumor responses and promoting tumor progression, metastasis, and angiogenesis [124–126]. M1 macrophages, in contrast, exhibit, a tumoricidal effect [127–129]. Monocytic MDSCs and TAMs share several characteristics, such as expression of the monocyte and macrophage markers F4/80 and CD115, as well as inducible expression of iNOS and ARG1 [123,124,130]. Accumulating evidence suggests that, upon entering tumor tissues, MDSCs may differentiate into TAMs, leading to elevated IL-10 production, inhibition of T cell responses, and promotion of angiogenesis [131]. However, the mechanism behind regulation of MDSC differentiation remains unclear [123,131,132].

Macrophages within the tumor microenvironment facilitate angiogenesis and extracellular-matrix breakdown and remodeling and promote tumor cell motility. There is a direct communication between macrophages and tumor cells that lead to invasion and egress of tumor cells into the blood vessels (intravasation). Thus, macrophages are at the center of the invasion microenvironment [133]. Mononuclear phagocytes are recruited in large numbers as primary monocytes from the circulation to diseased tissues, where they accumulate within ischemic/hypoxic sites terminally differentiating into inflammatory and tumor-associated macrophages or myeloid DCs. The cytokine MIF is over-expressed in tumors and is associated with tumor proliferation, angiogenesis and metastasis. Hypoxia, a hallmark feature of tumors, increases MIF expression from tumor cells. Inhibition of transcription and translation significantly decreased MIF production, suggesting that hypoxia-induced secretion of MIF is via an alternative pathway [134]. Hypoxia-mediated changes in mononuclear phagocyte gene expression and functional properties under different pathologic situations demonstrate that oxygen availability is a critical regulator of their functional behavior. Experimental evidence demonstrating that hypoxia modulates in primary monocytes the expression of a selected cluster of chemokine genes with a characteristic dichotomy resulting in the up-regulation of those active on neutrophils and the inhibition of those predominantly active on monocytes, macrophages, T lymphocytes, NK cells, basophils and/or DCs is reported. A negative regulatory role of hypoxia on monocyte migration is exerted through several alternative or complementary mechanisms and results in monocyte “trapping” within ischemic/hypoxic sites of diseased tissues. Data relative to the ability of hypoxia to differentially regulate in immature DCs (iDCs) the expression profile of genes coding for chemokines and chemokine receptors, the former being down-regulated and the latter up-regulated, thus promoting the switch from a proinflammatory to a migratory phenotype of iDCs by, respectively, reducing their capacity to recruit other inflammatory leukocytes and increasing their sensitivity to chemoattractants [135]. A partial overlap exists among mononuclear phagocytes at various differentiation stages in the expression of a cluster of hypoxia-responsive genes coding for regulators of angiogenesis, proinflammatory cytokines/receptors, and inflammatory mediators and implicated in tissue neo-vascularization and cell activation. Transcription pathways underlying hypoxia-regulated gene expression in monocytic lineage cells support a major role for the hypoxia-inducible factor-1 (HIF-1)/hypoxia responsive element (HRE) pathway in monocyte extravasation and migration to hypoxic sites and in the activation of monocyte/macrophage proinflammatory and immunoregulatory responses by hypoxia both *in vitro* and *in vivo*. Recent experimental evidence suggesting the requirement of additional transcription factors, such as nuclear factor-kappaB (NF-kappaB), Ets-1, CCAAT/enhancer binding protein-alpha/beta (C/EBPalpha/beta), activator-protein-1 (AP-1), and early growth response-1 (Egr-1), for hypoxic regulation of gene transcription in primary human monocytes and differentiated macrophages and indicative of the existence of both a positive and a negative O(2)-driven HIF-1-dependent feedback regulatory mechanism of hypoxia transcriptional response in primary monocytes [135].

Whether tumor-induced MDSC are normal cells halted in the intermediate stages of differentiation or whether they have diverged from the normal myeloid differentiation pathway and accumulated mutations is unclear. Direct comparisons of *in vitro* suppressive activity of splenic Gr1^+^CD11b^+^ cells from tumor-free mice *vs* tumor-bearing mice are not consistent. MDSC that are mononuclear are considered “monocytic” and typically are CD11b^+^Ly6G^+/−^Ly6C^high^, whereas those with multilobed nuclei are “granulocytic/neutrophil-like” and have a CD11b^+^Ly6G^+^Ly6C^low^ phenotype [136,137]. MDSC also vary in their content of immunosuppressive substances, with different populations containing arginase [138,139], inducible NO synthase [140], and/or additional ROS [138,140]. In cancer patients MDSC are typically CD11b^+^CD33^+^CD34^+^CD14^−^HLA-DR^−^ and can vary in their expression of CD15 and other markers [136,137]. The variation in MDSC phenotype is consistent with the concept that MDSC are a diverse family of cells that are in various intermediate stages of myeloid cell differentiation. MDSC are driven by tumor-secreted factors, and different tumors secrete different combinations of molecules. Therefore, MDSC phenotype will depend on the specific combination of factors within the tumor host. Because the myeloid population contains many different cell types and myeloid cell differentiation is a continuum of processes, MDSC may display diverse phenotypic markers that reflect the spectrum of immature to mature myeloid cells. This heterogeneity suggests that there may be no unique marker or combination of phenotypic markers that precisely defines MDSC, and that suppressive activity is the ultimate defining characteristic. It is also likely that as this population of cells is further studied additional subpopulations and markers will be identified.

MDSC accumulation and activation are driven by multiple factors, many of which are identified with chronic inflammation. Early studies demonstrated that the inflammation-associated molecules VEGF and GM-CSF were associated with the accumulation of MDSC [141], suggesting that inflammation might facilitate immune suppression [142]. However, it was not until the proinflammatory cytokines IL-1β [143] and IL-6 [144] and the bioactive lipid PGE_2_ [139] were shown to induce MDSC that the significance of the association with inflammation was appreciated. These later studies suggested that another mechanism by which inflammation promotes tumor progression is through the induction of MDSC that block immune surveillance and antitumor immunity, thereby removing barriers that could eliminate premalignant and malignant cells. MDSC are induced and/or activated by multiple proinflammatory mediators. MDSC accumulate in the blood, bone marrow, lymph nodes, and at tumor sites in response to proinflammatory molecules produced by tumor cells or by host cells in the tumor microenvironment. These factors include PGE_2_, IL-1β, IL-6, VEGF, S100A8/A9 proteins, and the complement component C5a.

The heterogeneity of MDSC also complicates finding a single strategy for eliminating the cells. Pathologically distinct tumors produce different arrays and quantities of proinflammatory factors that induce MDSC. As a result, there is phenotypic heterogeneity between MDSC induced by histologically distinct tumors. There is also phenotypic heterogeneity within the MDSC population induced within a single individual. This heterogeneity may require identifying and then specifically targeting the relevant proinflammatory mediator(s) for individual patients or for the specific type of tumor.

#### 3.1.5. From Tissue Microenvironment to Tumor Microenvironment

A widely held view posits, almost axiomatically, that clonal amplification of altered cells fuels carcinogenic process by increasing the likelihood that further genetic changes will occur in those dividing cells, towards the acquisition of a fully malignant phenotype [145]. Thus, according to this view, tumor promotion consists essentially, if not exclusively, in the clonal amplification of altered cells, which is *per se* sufficient to increase the risk for additional genetic hits, thereby fostering cancer development. While such interpretation might be theoretically appealing, it is pertinent to point out that early focal lesions resulting from tumor promotion (*i.e.*, polyps, papillomas, nodules) are generally not associated with the emergence of cellular sub-clones, as the hypothesis above would predict. It is also worth reiterating that promotion entails a mainly quantitative phenomenon of selective amplification and appears to be driven in many cases by physiological mechanisms involved in normal tissue turnover and/or reaction to injury [146]. In this context, the term focal lesion refers to a discrete collection of cells displaying a growth pattern and/or histological appearance, which are sufficiently distinct from that of the surrounding tissue resulting from the selective expansion of initiated/altered cells. According to this view, the focal nature of early lesions represents the critical end point of tumor promotion in that it forms the basis for the establishment of a new biological niche, with a fundamentally altered tissue architecture, which is generally referred to as the tumor microenvironment [82,147]. The emergence of the tumor microenvironment from the clonal growth of altered cells represents indeed *the quantum change* brought about by the tumor promotion phase in the natural history of cancer development. While the crucial role of the tumor microenvironment in controlling tumor progression and metastasis is now widely accepted, the formation of a specialized environment supporting CSC/CIC survival and growth (the CSC niche), its anatomical organization and the cellular and molecular mediators of these effects, are still under investigation [148]. The recent identification of human normal brain cells with self-renewal potential (reported as neural stem cells), that populate the subventricular zone, have contributed to the characterization of their niche and to the subsequent identification and characterization of brain tumor CSC/CIC and their niche. Brain tumor CSC/CIC are localized in a vascular niche that is supposed to provide factors promoting their self-renewal [149,150]. Perturbation of this niche, results in a compromised ability CSC/CIC to promote tumor growth within the brain [151,152]. These observations demonstrate the relevance of the vascular niche to tumor pathophysiology, and suggest the possibility to target the niche itself, and associated molecular events, to impinge on CSCs for therapeutic purposes [153,154].

Many signaling cascades controlling cell growth and proliferation are deregulated in cancers, resulting in excessive activation of downstream pathways, by signaling pathways that are not functional under nonpathologic conditions, and by alterations in gene expression patterns [4]. These situations may involve newly formed interactions between microenvironmental factors and tumor cells and between different microenvironmental factors [58,155–158]. These interactions constitute a fertile ground for the establishment of circular chains of tumor progression, enhancing events described as self-perpetuating cycles. Chemokine-driven self-perpetuating cycles operate in the progression of breast carcinomas in mice and humans. Monocyte chemoattractants CCL5 and CCL2 secreted by breast tumor cells may induce monocyte infiltration to the microenvironment of breast tumors. The resulting tumor-associated macrophages may secrete TNFα, which induces or up-regulates the secretion of several promalignancy factors from the tumor cells such as matrix metalloproteinases. TNFα also further up-regulates the secretion of CCL5 and CCL2, which drive the merry-go-round for another cycle [159]. It is not unlikely that similar cycles operate also in other types of cancer. A recent study indicated that a vicious cycle involving the CXCR3-CXCL10 axis and IFNγ operates in colorectal carcinoma progression [160]. CXCL10 secreted from CXCR3-expressing colorectal carcinoma cells promotes, by an autocrine mechanism, some progression-promoting functions in these tumor cells. CXCL10, at the same time, attracts CXCR3-expressing Th1 cells to the tumor site. The infiltrating Th1 cells secrete IFNγ, which, in addition to its immune functions, promotes the release of CXCL10 from IFNγ receptor-expressing colorectal carcinoma cells while up-regulating CXCR3 expression. This further promotes the capacity of the colorectal carcinoma cells to respond to CXCL10-mediated promalignancy functions. Self-perpetuating cycles involve multiple participants, each of which may serve as target for specific adjuvant treatment of metastasis. However, cancer cells are endowed with the capacity to bypass regulatory roadblocks or pathways by variety of mechanisms [161,162]. Every self-perpetuating cycle described above (and most others) involves numerous cellular interactions. Because the blocking of each interaction may be subject to a bypass strategy, we need to use multiple therapy modalities targeting most, if not all, components of such self-perpetuating cycles. A series of biochemical and metabolic changes are typically associated with the tumor microenvironment, being attributable, at least in part, to the altered blood and nutrient supply [83]. These changes can both induce and select for cell variants within the original focal cell population, setting the stage for the emergence and evolution of cell clones which represent the biological hallmarks of tumor progression [83,147,163–165]. Consistent with this interpretation, the analysis of multistage models of carcinogenesis has clearly documented that the long phase of tumor progression, leading from discrete focal lesions to the overt neoplastic phenotype, is a self-perpetuating process and does not depend on external manipulation, as it is the case for tumor promotion [146]. Thus, the unique microenvironment inside focal lesions, with its associated biochemical and metabolic alterations [83,147,165], appears to be sufficient to drive tumor progression.

### 3.2. Heterogeneity, Microenvironment and Metastasis

An understanding of cancer heterogeneity is incomplete without an analysis of metastatic tumors and disseminated tumor cells. Cancer is a systemic disease: malignant tumors shed large numbers of cells into the blood and lymph vessels, some of them developing in distant sites into metastases. Moreover, distant metastasis is responsible for the majority of cancer-related deaths, and, therefore, understanding the underlying biological mechanisms of it is of primary importance. The invasion/metastasis capability is closely related with cell motility and requires the cytoskeleton as a key component, which is also essential during mitoses. As malignancy criteria are mainly related with the phenotype of actively proliferating cells, it not surprising that metastatic deposits genetically match well differentiated areas of primary neoplasms, and that invasive areas (periphery of solid organ neoplasms and deep compartment of luminal organ tumors) show lower cellular turnover and higher incidence of genetic abnormalities [8,9,22,23,32]. These factors need attention when planning the evaluation of intratumoral heterogeneity and would include: detailed specification of sampling (intratumoral location, number of samples), combined evaluation of kinetic and genetic features to assess selective process, analysis of pathways at several steps to avoid confounding factors (redundancy and pleiotropism) [4,19,166]. These biological foundations will enable a better therapeutic design, using the heterogeneity to improve patient’s management.

There are two main models to explain the intratumoral heterogeneity: random process that would result in a patternless distribution or selective process that may be topographically linked and result in identifiable compartments including intraepithelial components. The malignancy-associated genetic instability can result in independent evolution in different tumor areas, regardless of the location (intraepithelial or invasive). Some so-called precancerous lesions show the genetic and kinetic features of established malignancies (clonal proliferation with accumulation of cooperative genetic abnormalities, and advantageous proliferation/apoptosis dysbalance) [19,25,31,167,168], questioning the appropriateness of the name. High-grade lesions are the most reliably diagnosed intraepithelial malignancies and progress through multiple morphological and molecular steps, which can and most likely include the acquisition of invasion/metastasis capability. As the order of these changes are not pre-established, they can be present well before the morphological evidence of malignancy [169], which reflect intratumoral heterogeneity and explain the presence of metastasis in cytologically low-grade lesions (multiple parallel pathways) that not necessarily progress through high-grade stage (linear pathways).

The connection between early neoplasm stages and metastasis is tumor heterogeneity and progression. Progression refers to the acquired capability of cell growth in surrounding or distant tissues, reflecting the acquisition of invasive capacities for intraepithelial lesions and metastatic capacities for invasive neoplasms. This capability reflects the interaction between tumor cells and the microenvironment. It would not be surprising to find similar control for the tumor cell capabilities in both steps (stromal invasion and metastasis) [4]. This progression would be related to the accumulation of genetic abnormalities and selective segregation of tumor cells with invasive capabilities, which are frequently topographically distributed [9,22,23,170]. Clinicopathological studies have demonstrated lymph node metastases associated with histologically intraepithelial malignancies (*i.e.*, breast, skin) [171,172]. Several intraepithelial foci showed more alterations than matched invasive foci, suggesting a more extensive genetic evolution for the former and supporting multifocality and independent clonal evolution of these coexistent carcinomas. The accumulation of genetic abnormalities in intraepithelial carcinomas is consistent with an advanced molecular stage and progression, along with topographic genetic heterogeneity [23,25,30,31,34,167]. Cancer cells are able to survive and proliferate only at specific secondary sites where there is an ideal environment that releases molecular mediators suitable for that type of cancer cells, still represents a main conceptual model of metastasis in modern cancer research [3,173–175]. Metastasis formation itself is a multi-step process that requires tumor cells to escape from the primary site, intravasate into the hematic or lymphatic circulation, migrate and extravasate into secondary organs [173,176]. Recent work has shed new light on the genetic, molecular and cellular basis of metastasis [177–179]. CSC/CIC might represent the unique sub-population of cells with the potential to successfully form metastasis in a distant organ [180,181]. Metastasis formation, however, is a rather inefficient process, mostly due to the need for a cancer cell to find a proper microenvironment for initiating tumor growth in a secondary organ. It has been proposed that in order to form metastases, primary tumors might produce factors that induce the formation of a suitable and appropriate environment in the organ where metastasis will be seeded. This has lead to the concept of the premetastatic niche, whereby a special, permissive microenvironment in secondary target organs is induced over distance by the primary tumor [182,183]. In this sense, there is a striking parallel between CSC/CIC maintenance and expansion in the primary tumor and formation of metastases in a distant organ. Both events require a particular niche or microenvironment and might share many cues promoting self-renewal ability, migration and invasion, resistance to apoptosis, and increased resistance to cytotoxic drugs. The first *in vivo* experimental evidence that metastatic seeding requires the formation of a niche was the discovery that VEGFR-1^+^ BMDC colonize target organs to form tumor-specific premetastatic sites, before the arrival of the metastatic tumor cells themselves [184]. In these sites BMDC express several hematopoietic markers, such as CD34, CD116, c-kit, Sca-1, as well as integrins (e.g., α4β1), chemokines and chemokine receptors (e.g., CXCL12/CXCR4), promoting either their homing to the target tissue or recruitment and attachment of tumor metastatic cells, or both.

What is known about the clonal relationship between tumor cells in primary tumors and metastatic sites? According to a traditional model of tumor development, tissue constraints constitute a major evolutionary bottle-neck in cancer evolution; thus, the acquisition of metastatic ability is considered to be the final step in tumor development, contingent on the acquisition of all of the other hallmarks of cancer [4–6]. This model implies that metastatic tumors should be genetically similar to the bulk of primary tumor cells.

Many studies that compared the genetic composition of primary tumors and secondary metastatic sites have found very close clonal relationships between the two in the majority of cases [185–187]. Similarly, analysis of gene expression profiles revealed very similar patterns between primary tumors and metastatic sites, a scenario highly unlikely for genetically divergent clones [188–190]. Another prediction from the linear model of tumor progression is that different metastases should display close clonal relationships among each other. Indeed, this prediction is supported by a recent study that compared the genetic composition of anatomically distinct metastatic lesions in 29 prostate cancer patients using SNP arrays and CGH [191]. In all cases, different metastatic lesions within the same patients demonstrated close clonal relationships, signifying monoclonal origin [191]. This demonstration of monoclonality of metastatic cancers is especially interesting given that primary prostate cancers are frequently multi-focal [192], and show substantial intra-tumor genetic heterogeneity [192,193].

While the evidence of the close genetic relationship between primary and metastatic tumors is compelling, some cases display dramatic divergence, challenging the model where acquisition of metastasis is considered to be the last step of tumor progression. Radically different patterns of allelic losses, indicative of a high degree of genetic divergence, have been reported between primary tumors and lymph node metastases in prostate cancers [186], and between primary tumors and asynchronous metastases in breast cancers [187]. Highly divergent clonal evolution was also evident in a subset of cases in CGH studies of primary tumors versus lymph node metastases in breast cancers and of primary tumors versus metastatic tumors in renal cell carcinomas [185]. A recent report, comparing sequences of primary tumors and metastases in lobular breast cancers, revealed multiple mutations present only in metastases and several other mutations with increased frequency in metastatic sites [194]. Some of these genetic changes result in higher incidence of apoptosis of tumor cells of dormant metastases (more than three fold higher) [195]. These data show that metastases remain dormant when tumor cell proliferation is balanced by an equivalent rate of cell death and suggest that angiogenesis inhibitors control metastatic growth by indirectly increasing apoptosis in tumor cells.

Important clues about the clonal evolution of tumors and the relationship between primary tumors and metastatic sites can be gained by the analysis of disseminated tumor cells. Because peripheral blood, bone marrow, and lymph nodes normally do not contain cells of epithelial origin, cells that have disseminated from primary tumors can be detected by the use of epithelial-specific markers, cytokeratins and epithelial cell adhesion molecule (EpCAM) [196–200]. Unexpected findings were reported by Klein and co-authors, who employed a PCR-based whole-genome amplification technique for the analysis of single disseminated tumor cells [201]. The authors demonstrated that disseminated tumor cells could be detected both in the bone marrow of patients and in mouse models of cancer at very early, pre-invasive stages of breast cancer development [169]. More support for the early dissemination of cancer cells came from CGH analysis of disseminated tumor cells from patients without overt metastases. These cells contained surprisingly few chromosomal aberrations [202], while higher-resolution LOH analysis did confirm the neoplastic origin of these cells and pointed to early events in tumor progression [203]. Interesting results were obtained for esophageal cancers, one of the most aggressive human carcinomas, where removal of primary tumors does not improve the chances of patient survival [204], suggesting the importance of relatively early metastatic spread. Lymph node and bone marrow metastases showed substantial divergence from primary tumors and among each other, suggesting parallel evolution shaped by microenvironment-specific selection forces. Interestingly, HER2 amplification in disseminated tumor cells was found to signify poor prognosis, whereas HER2 amplification in primary tumors had no prognostic value, further underlying the significance of divergent evolution between primary tumors and metastatic sites [205]. It should be noted, however, that since this analysis, based on the amplification of single-cell genomes, is associated with many technical caveats, critical interpretation of the data is highly warranted.

The obvious caveat of the studies of disseminated tumor cells is that the potential of any given disseminated single tumor cell to initiate a metastatic tumor is unknown. The mere fact that tumor cells survive at distant sites tells little about their ability to initiate a secondary tumor. Indeed, even normal epithelial cells were capable of extended survival in heterotopic sites in an animal model [206], and fetal cells are known to survive decades in women following pregnancy [207]. Also, most carcinomas display pronounced chromosomal instability, and, therefore, many disseminated cells might be a manifestation of “cell-to-cell heterogeneity”, *i.e.*, they are products of genomic instability without any substantial chance of clonal expansion. Testing the relevance of these disseminated cells to secondary metastasis formation is not trivial. The most compelling evidence comes from a mouse model of breast cancer where the growth of lung metastases paralleled that of the primary site from early points of tumor progression, and excision of mammary glands when primary tumors were still at the *in situ* stages did not prevent or delay the development of metastases [169]. Tumor cells that disseminated from early-stage tumors into the bone marrow were capable of lethal proliferation upon transplantation into lethally irradiated mouse recipients [169]. While these findings are compelling, their relevance to human cancer is not clear, especially considering that the genetic heterogeneity of disseminated cancer cells in mouse tumors appears to be less pronounced than that in human tumors.

Another group demonstrated that bone marrow aspirates from carcinoma patients contain disseminated tumor cells that are capable of proliferation in cell culture and that the extent of this proliferation is inversely correlated with patient survival [208]. CGH analysis of disseminated tumor cells from breast cancer patients confirmed their high divergence from primary tumors [209]. This study demonstrated substantially higher numbers of genomic abnormalities in disseminated tumor cells compared to the studies of Klein *et al*., despite similar staging of the tumors analyzed, suggesting that the ability of disseminated cells to form metastases is contingent on the acquisition of a large number of genetic alterations. Additional evidence for parallel clonal evolution comes from the detection of metastatic tumors with unknown primary origin, which constitute a significant fraction of clinical cases [210]. In addition, analysis of the growth kinetics of primary and metastatic tumors is inconsistent with metastatic spread being a late event [211].

Clearly, more studies are required to clarify the clonal relationship between primary and metastatic tumor cell populations, as this relationship could not only illuminate the evolutionary history of cancer development, but also create a more solid ground for therapeutic decision making. Currently, the majority of therapeutic decisions are being made based on the analysis of primary tumor specimens. Yet, this approach could only be justified in cases where the genetic compositions of primary and metastatic tumors are similar, especially when making decisions about emerging therapeutic approaches that target specific genetic changes. In addition, elucidating the issue of early *versus* late origin of metastasis-initiating cells is important for determining the usefulness of therapeutic approaches that target tumor cell invasion. The question of clonal heterogeneity within metastases remains unexplored, and, notably, clonal heterogeneity within primary tumors has limited direct therapeutic relevance, as in most cases primary tumors can be successfully removed by surgery, but this is most often not the case for metastatic outgrowths. Therefore, elimination of metastatic clones has to rely on adjuvant therapies, and it is likely that the success of these therapies will depend on the extent of the heterogeneity of these tumor cell populations.

## 4. Mechanisms Involved in Intra-Tumor Heterogeneity and Progression

Any type of cancer progresses through a relatively narrow evolutionary trajectory [212], “jackpot” mutations, which endow mutant clones with the ability to achieve selective sweeps, are likely to be very rare [4,16,18,19]. Most of the spontaneous mutations that occur are neutral or deleterious, and, while grossly disadvantageous mutations will be discarded, neutral or near-neutral mutations can undergo expansions due to genetic drift and can sometimes reach fixation in small tumors [60]. Tumor initiation and progression are dependent on rare, stochastic mutational events, and the chances of any given individual getting diagnosed with a particular cancer within a limited timeframe are relatively low, suggesting that cancers have to be clonal in origin, *i.e.*, they are likely to arise from a common progenitor. This consideration, supported by substantial experimental evidence, has led to a wide acceptance of the clonal origin of cancers [213,214], although there might be some rare exceptions in cases of hereditary cancers [215]. Rare mutations confer competitive advantage through cellular selection, resulting in clonal dominance. Even though most cancers start from a single mutated cell, mechanisms that constrain clonal evolution in populations of normal progenitor cells are no longer fully functional in tumors, allowing for an ongoing evolutionary process in populations of tumor cells [216]. Multiple rounds of proliferation, often counter-balanced by cell death, are required to produce macroscopic tumors, and genomic instability, observed in most cancers, is expected to constantly produce new mutations, which serve as raw material on which tumor evolution can work.

Moreover, spatial constraints that exist within a tumor can slow down the achievement of clonal dominance by substantially inhibiting competition among different tumor cell clones [9,10,22,23,34,166,217]. The importance of spatial constraints for the emergence and maintenance of clonal heterogeneity is supported by experimental observations in colon cancer as well as *in silico modeling* [218]. In addition to providing barriers to competition, spatial intra-tumor heterogeneity can support the co-existence of genetically distinct clones by affecting selective pressures. Most tumors represent at least partially structured habitats where individual cells within a tumor are experiencing differences in interactions with ECM, physical contact with other cells (both tumor and non-tumor), and gradients of oxygen, nutritional and growth factors, and metabolites. Therefore, tumor cells within different parts of tumors could be expected to experience different selective pressures, leading to the selection of different sets of mutations.

Mathematical modeling suggests that, even in the absence of substantial spatial heterogeneity, evolving pre-malignant tumors are likely to be characterized by quasi-steady states with the co-existence of multiple populations with different “strategies”. However, a clone that evolves toward invasive cancer will be capable of invading and destroying pre-malignant populations [219,220].

### 4.1. Hypoxia: Intratumor Variability and Influence on Metastasis

Hypoxia or oxygen deficiency is a salient feature of locally advanced solid tumors resulting from an imbalance between oxygen (O_2_) supply and consumption. Major causative factors of tumor hypoxia are abnormal structure and function of the microvessels supplying the tumor, increased diffusion distances between the nutritive blood vessels and the tumor cells, and reduced O_2_ transport capacity of the blood due to the presence of disease- or treatment-related anemia. Heterogeneities in tumor blood flow are associated with cyclic changes in pO_2_ or cyclic hypoxia. A major difference from O_2_ diffusion-limited or chronic hypoxia is that the tumor vasculature itself may be directly influenced by the fluctuating hypoxic environment, and the reoxygenation phases complicate the usual hypoxia-induced phenotypic pattern. Hypoxia induces a number of genes responsible for increased invasion, aggressiveness, and metastasis of tumors, including genes related with ECM interactions, migration, and proliferation [221]. Within a given tumor, there was an inverse correlation between regions of proliferation (Ki-67) and regions of hypoxia. The relationships between hypoxia and other biologic endpoints are complex, but, within a given tumor’s spatial relationships, they are in accord with known physiologic principles. Necrosis, proliferation, and blood vessel distribution cannot predict the level or presence of hypoxia in an individual tumor [222]. The exposure of endothelial cells to cycles of hypoxia/reoxygenation led to accumulation of HIF-1alpha during the hypoxic periods and the phosphorylation of protein kinase B (Akt), extracellular regulated kinase (ERK) and endothelial nitric oxide synthase (eNOS) during the reoxygenation phases. Cyclic hypoxia, as reported in many tumor types, as a unique biological challenge for endothelial cells that promotes their survival in a HIF-1alpha-dependent manner through phenotypic alterations occurring during the reoxygenation periods [223].

**Figure 4:**
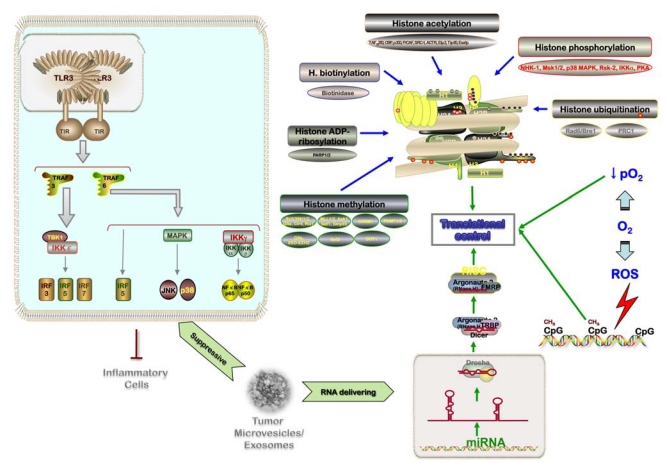
Differential gene regulation in neoplasms. This process is related with extracellular suppressive mechanisms and RNA delivering through exosomes, hypoxic microenvironment, and epigenetics (methylation and histone modifications).

Histopathological examination of solid tumors frequently reveals pronounced tumor cell heterogeneity, often demonstrating substantial diversity within a given tumor. The molecular mechanisms underlying the phenotypic heterogeneity are very complex with genetic, epigenetic and environmental components, such as shortage in oxygen. Hypoxic tumors appear to be poorly differentiated. Increasing evidence suggests that hypoxia has the potential to inhibit tumor cell differentiation, thus playing a direct role in the maintenance of CSCs, also blocks differentiation of mesenchymal stem/progenitor cells, a potential source of tumor-associated stromal cells. It is therefore likely that hypoxia may have a profound impact on the evolution of the tumor stromal microenvironment, facilitating tumor progression. Hypoxia may help create a microenvironment enriched in poorly differentiated tumor cells and undifferentiated stromal cells. Such an undifferentiated hypoxic microenvironment may provide essential cellular interactions and environmental signals for the preferential maintenance of CSCs [224]. Hypoxia greatly influences cellular phenotypes by altering the expression of specific genes, makes the tumors more aggressive (Figure 1), and is an important contributor to intra- and inter-tumor cell diversity as revealed by the pronounced but non-uniform expression of hypoxia-driven genes in solid tumors [225]. Hypoxic tumor cells lose their differentiated gene expression patterns and develop stem cell-like, immature or dedifferentiated phenotypes. Hypoxia-induced dedifferentiation will not only contribute to tumor heterogeneity but could also be one mechanism behind increased aggressiveness of hypoxic tumors. A state of hypoxia is a universal characteristic of the microenvironment of solid tumors. Growing evidence indicates that hypoxia plays a critical and fundamental role in metastasis formation [226]. Hypoxia drives tumor progression by influencing both the tumor cells as well as the tumor microenvironment (Figure 4). It increases genomic instability and heterogeneity in tumor cells and selects resistant tumor variants. It also alters the expression of several genes, among them hypoxia-inducible factor 1, a transcription factor that regulates a large variety of key genes controlling cell survival, migration, and angiogenesis to name just a few [227,228]. Hypoxia and hypoxia-inducible factor 1 mediate progression-promoting or progression-restraining effects on tumor cells and on the microenvironment. These activities involve, on the one hand, regulation of genes that drive tumor progression [227,228], and, on the other hand, the induction of genes that may cause the opposite effect. (e.g., cell death). By surviving and propagating under hypoxia, resistant tumor cells further aggravate the state of tumor hypoxia. This, in turn, increases genomic instability and further stabilizes and activates hypoxia-inducible factor 1. This vicious cycle drives alterations of gene expression patterns in tumor cells and thereby enhances tumor progression.

Hypoxia promotes each step of the metastatic cascade, including the promotion of angiogenesis and lymphangiogenesis, the induction of changes in vascular integrity and permeability (that facilitate tumor cell intravasation and extravasation), as well as cell proliferation [4]. Most of these mechanisms are regulated by HIF-induced VEGF action, which become connected with the coexpression through pVHL and might enable tumor progression and metastatic dissemination by autocrine receptor stimulation [229]. Hypoxia greatly influences cellular phenotypes by altering the expression of specific genes, makes the tumors more aggressive, and is an important contributor to intra- and inter-tumor cell diversity as revealed by the pronounced but non-uniform expression of hypoxia-driven genes in solid tumors [225]. Intratumoral hypoxia is an independent indicator of poor patient outcome and increasing evidence supports a role for hypoxia in the development of metastatic disease [225]. Studies suggest that the acquisition of the metastatic phenotype is not simply the result of dysregulated signal transduction pathways, but instead is achieved through a stepwise selection process driven by hypoxia [230–232]. Through upregulation of urokinase-type plasminogen activator receptor (uPAR) expression [233], hypoxia enhances proteolytic activity at the invasive front and alters the interactions between integrins and components of the ECM, thereby enabling cellular invasion through the basement membrane and the underlying stroma [229,234,235]. Cell motility is increased through hypoxia-induced hepatocyte growth factor (HGF)-MET receptor signaling, resulting in cell migration towards the blood or lymphatic microcirculation. Hypoxia-induced vascular endothelial growth factor (VEGF) activity also plays a critical role in the dynamic tumor-stromal interactions required for the subsequent stages of metastasis [236]. HIF1-induced VEGF promotes angiogenesis and lymphangiogenesis, providing the necessary routes for dissemination, induces changes in vascular integrity and permeability that promote both intravasation and extravasation, and is essential for cell proliferation in metastatic lesions.

The important steps that enable metastasis are reversible, and therefore cannot be explained solely by irreversible genetic alterations, indicating the existence of a dynamic component to human tumor progression, in particular a regulatory role for the tumor microenvironment [178]. Hypoxic tumor cells lose their differentiated gene expression patterns and develop stem cell-like, immature or dedifferentiated phenotypes [102]. Hypoxia-induced dedifferentiation will not only contribute to tumor heterogeneity but could also be one mechanism behind increased aggressiveness of hypoxic tumors. The loss of epithelial characteristics (*i.e.*, lack of intercellular adhesion molecules) results in breakdown of epithelial-cell homeostasis, correlates with the acquisition of a migratory and stem cell phenotype that leads to progression, and it is regulated by CXCR4-CXCL12 [237]. The epithelial to mesenchymal transition is considered to be a crucial event in malignancy [238]. Tumors are morphological and functionally heterogeneous and segregate tumor cell with different capabilities: individual tumor shows distinct sub-areas of proliferation and cell-cycle arrest, differentiation, cell adhesion and dissemination, some of them determined by topography [4,8–10,16,22,23,32,81,217,239]. Supporting this concept, topographic analysis of HIF1 and stem cell markers in follicular thyroid neoplasms has shown direct correlation. Hypoxia facilitates disruption of tissue integrity through repression of E-cadherin expression, with concomitant gain of N-cadherin expression, which allows cells to escape anoikis [4]. Neoplastic transformation evolves over a period of time involving the phenotypic progression of tumor cells along with the interaction of the initiated cell with its microenvironment. Tumor cell dissemination is the prerequisite of metastases and is correlated with loss of epithelial differentiation and the acquisition of a migratory phenotype through a stepwise, irreversible accumulation of genetic alterations [177,180,238]. The molecular mechanisms led by hypoxia explain the general phenotype associated with progression (metastatic capability): spindle-shaped cellularity, necrosis and stem cell phenotype. CXCR4-CXCL12 and hypoxia play a central role on the metastatic process (stromal interaction and digestion, angiogenesis), maintaining stem cell features in the tumor cells. Metastasis is considered the final step in malignancy that results from selected aspects of the complex tumor-progression process. However, this capability can be acquired early during the natural history of neoplasms and has a complex molecular regulation, for which the interaction tumor cell-stroma is essential. The chemokine axis CXCR4-CXCL12 has demonstrated a central role in this mechanism [240,241], involving both endothelial cells interactions and expression of metalloproteinases [229,233,242]. The central role of hypoxia in tumor progression-metastasis would also explain the general phenotype associated with progression (metastatic capability): spindle-shaped cellularity, necrosis and stem cell phenotype. CXCR4-CXCL12 and hypoxia play a central role on the metastatic process (stromal interaction and digestion, angiogenesis), maintaining stem cell features in the tumor cells. Among the chemokines involved in tumor metastasis, the CXCL12 (also known as SDF-1 or stromal-derived factor)/CXCR4 axis plays a critical role in stem cell migration. Activation of CXCR4 induces motility, chemotactic response, adhesion, secretion of MMPs and release of angiogenic factors, such as VEGF-A. Interestingly this premetastatic niche, like the normal niche, is characterized by the presence of specific ECM proteins, such as fibronectin, a ligand for α4β1 expressed on VEGFR-1^+^ cells. Thus, it appears that in order to survive at distant sites, disseminating tumor cells need to recreate a supportive microenvironment similar to the one formed in the primary tumor. One efficient way to do it is by directing BMDC or immune/inflammatory cells to these distant sites of future metastasis [243]. The chemoattractant proteins S100A8 and S100A9 were the first factors shown to instruct the formation of the premetastatic niche [183,244]. Additional studies are needed to further elucidate the biological mechanisms involved in the formation of this premetastatic niche, in particular regarding the identification of the molecular mechanism responsible for the development of clinically relevant metastases from the initial seeds.

### 4.2. Regulation of Gene Expression: Exosomes and Epigenetics

The phenotypic heterogeneity in neoplasms is mainly related with differential gene expression. Gene expression analysis is becoming a useful tool for a better definition of neoplasms at diagnostic, prognostic and predictive levels. The identification of predictive markers of these features will help classifying neoplasms and stratifying patients for a better management. However, the nature and biological meaning of these gene expression markers is not always clear: origin of the tested RNA, mechanism of RNA transference, utility for subclassification of neoplastic lesions.

Gene expression analysis is becoming a useful tool for a better definition of neoplasms at diagnostic, prognostic and predictive levels [245–249]. *Tumor hypoxia* induces changes in the proteome and genome of neoplastic cells that enable the cells to overcome nutritive deprivation or to escape their hostile environment. The selection and clonal expansion of these favorably altered cells further aggravate tumor hypoxia and support a self-perpetuating circle of increasing hypoxia and malignant progression while concurrently promoting the development of more treatment-resistant disease [250]. The Met tyrosine kinase, a high affinity receptor for hepatocyte growth factor (HGF), plays a crucial role in controlling invasive growth and is often overexpressed in cancer. Hypoxia activates MET protooncogene transcription, amplifies HGF (hepatocyte growth factor) signaling, and synergizes with HGF in inducing invasion; the proinvasive effects of hypoxia are mimicked by Met overexpression (Figures 2 and 4). Hypoxic tumor areas overexpress Met and the inhibition of Met expression prevents hypoxia-induced invasive growth. These data show that hypoxia promotes tumor invasion by sensitizing cells to HGF stimulation, providing a molecular basis to explain Met overexpression in cancer [251]. There is also a link between a specific group of microRNAs and hypoxia, a key feature of the neoplastic microenvironment: A significant proportion of the hypoxia-regulated microRNAs (HRMs) are also overexpressed in human cancers, suggesting a role in tumorigenesis, affecting important processes such as apoptosis, proliferation and angiogenesis. Several HRMs exhibit induction in response to HIF activation [252].

The gene expression signature also reflects the *metastatic potential* in both cell lines and tumor samples [253–257]. Some general conclusions have been drawn in this respect. First, the expression profile of low metastatic subline is distinct from that of the high metastatic subline. Metastases of the low metastatic subline closely resembled the profile of the low metastatic primary tumor, and did not “switch over” to the high metastatic gene expression profile. Thus, heterogeneity exists in this cell line/tumor, but may not be apparent as the signature reflects the majority of the tumor cells. The low metastatic subline is capable of spawning metastases, albeit at a lower rate, and its gene expression profile is therefore of significant interest. Second, the gene expression profile of the sublines as *in vitro* cultures is very distinct from the same sublines as primary tumors. While the primary tumors are a mixture of tumor cells, stromal cells, endothelial cells, etc, the contribution of these cells to the observed gene expression profiles should be minimal. Thus, the differences are thought to reflect changes in the tumor cells in response to the *in vivo* microenvironment. Third, comparable numbers of genes were “turned on” and “turned off” in the more highly metastatic tissues. We think about metastasis as the acquisition of traits, but the data remind us to pay equal attention to the loss of growth and differentiation-controlling genes. Many of the differentially expressed genes fall into the category of “usual suspects” for metastasis. These include the overexpression of osteopontin and ECM genes in the more metastatic tissues, and the loss of thrombospondin 1 and members of the Nm23 family [247]. Eight metastasis-suppressor genes that reduce the metastatic propensity of a cancer cell line *in vivo* without affecting its tumorigenicity have been identified. These affect important signal-transduction pathways, including mitogen-activated protein kinases, RHO, RAC and G-protein-coupled and tyrosine-kinase receptors [255]. Other genes are unexpected and, if validated functionally, could shed new light on the mechanisms of metastasis.

Cancer cells communicate with the environment through delivery of surface proteins, release of soluble factors (growth factors and cytokines), and sophisticated nanovehicles (exosomes) for establishment of invasive tumor growth. A central question in molecular studies is to determine the *cellular origin of the target mRNA*. Two biological aspects are essential for this process: how the tumor mRNA gets there and the functionality of this mRNA. This is most relevant in locations where the tumor cells are scanty and the expression profile support higher tumor cell load [258]. Most of the cases of malignant melanomas do not show enough number of superficial tumor cells to explain the positive findings in superficial samples [259]. In this context, the RNA cannot be directly provided by the tumor cells but by keratinocytes through a transfer process similar to what happens with melanin. *Membrane vesicles* derived from both tumor and host cells have recently been recognized as new candidates with important roles in the promotion of tumor growth and metastasis [260]. The transfer of membrane components between donor and acceptor cells has been described “trogocytosis” (from Greek “trogo”, meaning “gnaw” or “bite”). Two forms of membrane transfer (trogocytosis) have been described: via nanotubes or via membrane vesicles [261]. The biogenesis of membrane vesicles essentially distinguishes exosomes from shedding microvesicles and apoptotic blebs (Figures 2 and 4). Microvesicles (exosomes) containing mRNA and miRNA can be taken up by normal host cells, such as keratinocytes or endothelial cells, and translated by recipient cells [262]. Exosomes are specialized membranous nano-sized vesicles derived from endocytic compartments that are released by many cell types; they are formed by the inward budding of multivesicular bodies (MVBs) and are released from the cell into the microenvironment following the fusion of MVBs with the plasma membrane [263]. Intercellulars communication occurs in part through constitutive exocytosis, regulated exocytosis, or release of intraluminal vesicles, and is modulated by small Rab GTPases, the master regulators of vesicle traffic. Rab GTPases are implicated in regulated exocytosis and have showed a unique role for Rab27B in invasive tumor growth. Emerging evidence indicates that various exocytic routes are implemented by cancer cells to relay crucial information for fostering growth, migration, and matrix degradation [264]. Tumor-derived microvesicles therefore serve as a means of delivering genetic information and proteins to recipient cells in the tumor environment, and the subset of tumor suppressive microvesicles are key elements of tumor-related suppression mechanisms by accumulations of adenosine-producing regulatory T-cells in the tumor microenvironment and expression of toll-like receptors on the tumor cell surface [265]. It has been suggested that microvesicles shed by certain tumor cells hold functional messenger RNA (mRNA) that may promote tumor progression. Purified exosomes contain functional microRNAs (miRNAs) and small RNA, but detected little mRNA. Exosomes are specialized in carrying small RNA including the class 22–25 nucleotide regulatory miRNAs. Both the evidence provided from studies on exosomes and the lack of the expected phenotypic changes in keratinocytes from the expression of putative melanoma markers in the study support a transfer of predominant short RNA rather than functional mRNA [259,262]. The discovery of extracellular miRNAs (including miR-21), existing either freely or in exosomes in the systemic circulation, has led to the possibility that such molecules may serve as biomarkers for ongoing patient monitoring [266]. There is increasing evidence that miRNAs have potential not only to facilitate the determination of diagnosis and prognosis and the prediction of response to treatment, but also to act as therapeutic targets and replacement therapies [267,268]. The biological heterogeneity in neoplasm and precancerous lesions would potentially make more difficult these assessments [34,269,270]. We only have to consider that deep tumor compartments provide more accurate prognostic information in melanomas and dermal compartments rather than the junctional compartments better define dysplastic melanocytic lesions.

The biologic heterogeneity of intraepithelial and invasive malignancies is well known at the morphologic, kinetic and genetic levels (Figure 1), an issue that has not been addressed in this paratumoral gene expression analysis and should warrant future studies. The gene expression markers should be evaluated in the appropriate biological context. Gene expression profiles are determined by a gene regulatory network comprising regulatory core of genes represented most prominently by transcription factors and miRNAs, which influence the expression of other genes, and a periphery of effector genes that are regulated but not regulating [271,272]. Most studies do not differentiate between these two essential groups, which can be also useful in selecting surrogate markers for a given condition [4,271]. There is a general concept to keep in mind for gene expression analyses: the amount of information is overwhelming and the number of variables included in the studies significantly outnumbers the cases. In this scenario, the significant variables can be the result of a statistical selection rather than the expression of a biologically significant process for a particular neoplasm. Supporting this aspect, significant variables frequently include genes of the general metabolic activation associated with the neoplastic transformation [4,5], rather than tissue- or differentiation-specific gene variables.

*Epigenetic* dysregulation is central to cancer development and progression (Figure 4) [273]. The best-known epigenetic marker is DNA methylation. This dysregulation includes hypomethylation leading to oncogene activation and chromosomal instability, hypermethylation and tumor suppressor gene silencing, and chromatin modification acting directly, and cooperatively with methylation changes, to modify gene expression [274]. The initial finding of global hypomethylation of DNA in human tumors was soon followed by the identification of hypermethylated tumor-suppressor genes, and then, more recently, the discovery of inactivation of microRNA (miRNA) genes by DNA methylation. (I) DNA methylation occurs in a complex chromatin network and is influenced by the modifications in histone structure that are commonly disrupted in cancer cells [273]. Three mechanisms have been proposed to explain the contribution of *DNA hypomethylation* to the development of a cancer cell: generation of chromosomal instability, reactivation of transposable elements, and loss of imprinting. Undermethylation of DNA can favor mitotic recombination, leading to deletions and translocations [275,276], and it can also promote chromosomal rearrangements. This mechanism was seen in experiments in which the depletion of DNA methylation by the disruption of DNMTs caused aneuploidy [277]. Hypomethylation of DNA in malignant cells can reactivate intragenomic endoparasitic DNA, such as L1 (long interspersed nuclear elements), and Alu (recombinogenic sequence) repeats [278]. These undermethylated transposons can be transcribed or translocated to other genomic regions, thereby further disrupting the genome. (II) *Hypermethylation of the CpG-island promoter* can affect genes involved in the cell cycle, DNA repair, the metabolism of carcinogens, cell-to-cell interaction, apoptosis, and angiogenesis, all of which are involved in the development of cancer [279]. Hypermethylation occurs at different stages in the development of cancer and in different cellular networks, and it interacts with genetic lesions. Such interactions can be seen when hypermethylation inactivates the CpG island of the promoter of the DNA-repair genes *hMLH1, BRCA1, MGMT* (O^6^-methylguanine–DNA methyltransferase), and the gene associated with Werner’s syndrome (*WRN*) [280–282]. In each case, silencing of the DNA-repair gene blocks the repair of genetic mistakes, thereby opening the way to neoplastic transformation of the cell. (III) Hypermethylation of the CpG islands in the promoter regions of tumor-suppressor genes in cancer cells is associated with a particular combination of histone markers: deacetylation of histones H3 and H4, loss of H3K4 trimethylation, and gain of H3K9 methylation and H3K27 trimethylation [27,283]. The presence of the hypo-acetylated and hypermethylated histones H3 and H4 silences certain genes with tumor-suppressor–like properties, such as *p21^WAF1^*, despite the absence of hypermethylation of the CpG island. In human tumors generally, modifications of histone H4 entail a loss of monoacetylated and trimethylated forms [284]. These changes appear early and accumulate during the development of the tumor [284]. (IV) Short, 22-nucleotide, non-coding RNAs that regulate gene expression by sequence-specific base pairing in the 3′ untranslated regions of the target mRNA are called miRNAs. The result is mRNA degradation or inhibition of translation [285]. DNA hypermethylation in the miRNA 5′ regulatory region is a mechanism that can account for the down-regulation of miRNA in tumors [286]. In colon-cancer cells with disrupted DNMTs, hypermethylation of the CpG island does not occur in miRNAs [286]. The methylation silencing of *miR-124a* also causes activation of the cyclin D–kinase 6 oncogene (*CDK6*), and it is a common epigenetic lesion in tumors [286].

## 5. Clinical Implications

### 5.1. Diagnosis: Sampling and Genetic Targets

The application of the so-called hallmarks of cancer in oncological pathology leads to consideration of the molecular test requirements (Molecular Test Score System) for reliable implementation; these requirements should cover biological effects, molecular pathway, biological validation, and technical validation [4]. Sensible application of molecular markers in tumor pathology always needs solid morphological support.

Additionally, any new test should be validated against the accepted standard (specificity/sensitivity), demonstrate improvements of patient’s management, and should provide a biologic meaning for its application. These requirements could be considered as implementation phases: The first requirement is normally met on the initial design, the second requirement would be expected in any successful implementation, while the third is more difficult to prove. Any new definition should be biologically meaningful and would incorporate core elements in tumor biology (in particular genetic and kinetic correlates). Examples include the PAX8/PPARγ fusion gene described in follicular thyroid carcinomas and adenomas, or RET/PTC fusion genes reported in papillary thyroid carcinomas and Hashimoto’s thyroiditis. Apart from the lack of general interest, the scarcity of definitive publications on clonal heterogeneity can be attributed to the substantial technical challenges faced by researchers who want to study genetic heterogeneity in human cancers. Several approaches have been used to study genetic heterogeneity, which need to address the issue of inadequate sampling and technical caveats arising from the frequent necessity to analyze limited amounts of starting material. It is useful to divide the approaches to study clonal heterogeneity into focused and genome-wide methods.

#### 5.1.1. Sampling Issues

One of the obvious challenges of studying clonal heterogeneity in human malignancies is the issue of sampling. One aspect of this problem is the availability of representative biopsies. Core biopsies, obtained for diagnostic purposes, sample only small regions of tumors, and, therefore, are not likely to be informative about the clonal composition of each tumor as a whole. Thus, adequate analysis of intra-tumor clonal heterogeneity often relies on access to post-surgical samples from different regions of tumors [9–11,22,23,30,217].

Sample size is another important issue [17,18,28]. When a tumor specimen is too large, most methods will only provide an average picture, usually reflecting the dominant clone. Thus, potential heterogeneity would be missed: while large numbers of cells pooled will quench the “noise”, signal from minor subpopulations will be lost. The exception is deep sequencing of whole genomes, which allows for the detection of mutant alleles present in a small fraction of tumor cells [194]. On the other end of the spectrum, many of the analyses suitable for the characterization of clonal heterogeneity can be performed with starting material of just single cells [201]. Focusing on single cells, however, brings about the problem of dealing with cell-to-cell variability, where some of the distinguishable variants have no chances of clonal expansion and thus constitute evolutionary “dead ends”. This problem of biological noise can potentially be dealt with by analyzing larger numbers of individual cells, but scaling up the analysis will inevitably drive up the cost and labor required, making these studies impractical. Moreover, single-cell analysis suffers from increased vulnerability to technical caveats related to the necessity to amplify single genomes prior to the analysis (discussed below).

Therefore, with respect to sample size, there is a need to balance signal-to-noise ratios. Many studies achieve this balance by focusing on relatively small regions of tumors, ideally representing morphologically distinct units that are isolated by microdissection. The validity of this approach is supported by the observation of the clustering of populations that differ in gene expression [287], as well as genetic composition [218]. However, sampling small regions of tumors can still miss relatively small patches of genetically distinct cells, unless large numbers of samples are taken from the individual tumors [17,18,28]. In addition, if genetically distinct clones are intermixed within small anatomically distinct units, such heterogeneity will be missed due to pooling of cells.

Another approach to sampling is based on the analysis of phenotypically distinct subpopulations after singling them out by FACS, by immunobead separation or by morphological appearance and microdissection. For example, analysis of the genomic composition of CD44^+^CD24^−^ and CD44^−^CD24^+^ breast carcinoma cells has revealed the existence of unexpected clonal divergence between the two populations in some tumor samples [288]. Obviously, this approach can be valid only for the cases when phenotypic differences can be related to differences in genetic composition, and genetic heterogeneity within the populations will be missed.

#### 5.1.2. Focused Approaches

In focused approaches, analysis is limited to a particular genetic locus or a set of loci. The advantage of focused approaches is that they allow for the analysis of specific genes that have been implicated as drivers of tumor progression. Current knowledge of cancer genetics permits focusing on genetic lesions specific for types and subtypes of a particular cancer. The obvious disadvantage of these methods is that limiting analysis to a few loci will miss other potentially important differences and will likely underestimate the extent and patterns of clonal heterogeneity.

Most of the published reports on intra-tumor clonal heterogeneity have relied on the detection of allelic imbalances. Most commonly, this analysis is performed on microdissected tumor samples to ensure lack of contamination by normal cells and to provide adequate sample sizes. The DNA is then isolated from the captured cells, and PCR is performed using primers against microsatellite markers specific to regions that have previously been characterized as being frequently altered in the particular type or subtype of cancer under study. Comparison to controls, germline samples from the same patients, allows for the detection of alleles that have been lost somatically. Although obtaining data is relatively simple, straightforward interpretation is obscured by multiple technical caveats, such as frequently poor correlation between chromosomal deletion/amplification and under/overrepresentation of relevant microsatellite markers [289]. Therefore, data obtained by analysis of allelic imbalances often requires confirmation by independent techniques.

Studies of clonal heterogeneity may rely on the detection of amplification of specific chromosomal regions by fluorescent *in situ* hybridization (FISH) or immuno-FISH (FISH coupled with immunofluorescence) analysis [42,288]. In FISH analysis, fluorescently labeled BAC (bacterial artificial chromosome) probes against regions amplified in the particular cancer type, as well as control probes for centromeric regions, are hybridized to cancer cells or tissue sections. Combination with immunofluorescence provides the advantage of focusing on specific subtypes of tumor cells. FISH analysis is less vulnerable to artifacts than are other approaches, such as analysis of allelic imbalances; thus, it can be the approach of choice for the validation of differences initially detected by other means. Disadvantages include its labor-intensiveness, as large numbers of cells need to be analyzed in order to generate statistically meaningful data. Also, although potentially suitable for the analysis of chromosomal deletions, detection of amplified regions is more practical.

Clonal heterogeneity can be assessed by sequencing specific genes known to be frequently mutated in a particular cancer [218,290]. This analysis usually involves microdissection and PCR amplification of the samples. The major advantage of sequencing is that it allows for focusing on specific “driver” genes; thus, any heterogeneity will most likely be of functional importance. Intensive studies in the field of cancer genetics, and recent sequencing of cancer genomes and allowed the identification of cancer-type-specific subsets of genes that “drive” cancer progression [288,291,292]. Therefore, it should be feasible to analyze samples for sets of genes causally implicated in a given cancer. Also, instead of the detection of specific nucleotide changes, bisulfite sequencing of specific alleles can be used to detect heritable methylation changes either in promoters of “driver” genes or in a larger set of “passenger” sites, which can be used as markers to trace clonal expansion patterns [293].

Lymphoid malignancies sometimes present another unique opportunity for focused clonality analysis. Early steps of development of B and T cells involve step-wise rearrangements of immunoglobulin (Ig) and T-cell receptor (TCR) genes, respectively. Germline loci for Ig and TCR genes contain multiple alternative gene segments, encoding for gene domains, that are randomly chosen for rearrangements to generate thousands to millions (depending on the gene) potential mature rearranged products distinguishable by PCR. While, in most cases, a given leukemia or lymphoma arises from a cell with completed rearrangement, and, thus, all of the cells in the malignancy start with an identical rearrangement, the immunogene loci often undergo further rearrangements through recombination or somatic hypermutation, providing a pattern that can be used to analyze evolutionary relationships among distinct clones [294,295]. Combining PCR and deep sequencing appears to be an especially promising way to analyze this, as it allows for the detection of rare subclones without *a priori* knowledge of the products of rearrangements [155,296]. One potential drawback is that the subclonal changes might have no effect on fitness, i.e., they are evolutionarily neutral. However, the approach is still useful given that it allows for establishing evolutionary relationships between primary disease and relapses [297,298], and permits tracing the evolutionary history of leukemias.

#### 5.1.3. Genome-Wide Approaches

Instead of focusing on particular loci, whole genomes can also be analyzed in clonal heterogeneity studies. An obvious advantage of these approaches is that their success is not contingent on *a priori* knowledge; therefore, potentially important differences, missed by focused approaches can be detected. The major disadvantages of these methods are that the functional impact of detected differences is often unclear and that the clonal differences might be evolutionarily neutral. In addition, many genome-wide approaches suffer from limited resolution.

Karyotypic analysis based on chromosomal banding was frequently used in early reports on clonal heterogeneity [299,300]. In this technique, tumor cells are subjected to short-term culture followed by fixation and staining, which reveals a pattern of chromosomal banding in metaphase chromosomes. This method allows for the detection of large chromosomal abnormalities, but it has several pitfalls. Karyotypic analysis requires culturing the cells, which may lead to preferential outgrowth of selected tumor cell subpopulations, changing the representation of the original tumor. It is also labor-intensive and has low resolution.

More recent publications usually rely on comparative genomic hybridization (CGH) to detect chromosomal aberrations. DNA from tumor and normal cells is differentially labeled with fluorescent probes and, in the classical form of the assay, hybridized to normal metaphase chromosomes. Fluorescent signal is then analyzed using specialized software, revealing regions of chromosomal losses and gains. CGH is one of the most widely used techniques in studies of intra-tumor clonal heterogeneity. It provides unbiased coverage of the whole genome. However, hybridization to metaphase chromosomes gives limited resolution, as small regions of deletion and amplification can be missed. Instead of metaphase chromosomes, differentially labeled DNA can be hybridized to genomic arrays, allowing for the detection of smaller-scale allelic imbalances up to the single-nucleotide level using single nuclear polymorphism (SNP) arrays [288]. Notably, analysis of chromosomal imbalances can be performed on the level of specific chromosomes (using chromosomal arrays) rather than on a whole-genome scale. Finally, clonal heterogeneity can potentially be detected by the analysis of DNA content, where tumor cells are stained with DNA-labeling dye and subjected to flow cytometric analysis (FACS). This approach reveals ploidy status and the distribution of cells in different phases of the cell cycle, and, in cases of large differences, allows for the detection of populations with distinct aneuploidy based on their shift from normal ploidy peaks [13,301–303]. The advantages of this method are that sampling issues are not much of a problem (discussed below) and that it is capable of resolving even highly intermixed subpopulations. However, its resolution is limited to the detection of clones that differ substantially in DNA content. Therefore, the method provides limited molecular insights unless distinct subpopulations are sorted and subsequently analyzed with more sensitive methods.

#### 5.1.4. Issues of Quantity and Quality

Many methods of tumor heterogeneity analysis rely on microdissection of small, anatomically distinct sections of tumors from frozen or archived tissues. Microdissection allows for focusing the analysis on small, morphologically homogeneous populations and avoids contamination with non-malignant cells. However, the technique leads to increased DNA damage and thus reduced quality. The issue of DNA quality is especially pronounced in the analysis of archived formalin-fixed paraffin embedded tissues. Clonality remains as one of the most important hallmarks of neoplasms, but its analysis require careful test designs. The core of comprehensive clonality assays would include a complementary approach comprising the evaluation of structural abnormalities of key oncogenes and tumor suppressor genes (mutations, allelic imbalance) that control essential acquired capabilities in neoplasms. Pathway independent tests based on X-chromosome inactivation and the evaluation of epigenetic alterations such as methylation levels have a more restrictive application in female and specific tumor types with high imprinting levels, respectively. This approach would resolve the patch size problem that is frequently associated with clonal expansions, which can be highlighted in single marker analyses.

Due to the low amount of starting material, many tumor heterogeneity analysis methods, especially those involving whole-genome analysis, rely on prior amplification of whole genomes. Several efficient methods for whole-genome amplification, relying either on PCR-based or isothermal DNA amplification, have been developed over the last decades. However, whole-genome amplification produces only a representation, not a replica, of the genome, so, inevitably, representation bias is introduced. This bias might be more pronounced in PCR-based techniques due to different efficiencies of amplification of fragments of different sizes and nucleotide composition. However, even isothermal, linear amplification techniques have been shown to suffer from substantial representation bias [304]. The issue of representation bias is most pronounced when the starting material is small (<10 cells) [18,28], culminating at the single-cell level. To some extent, bias issues can be reduced by using identically manipulated control samples. However, acquiring robust data often requires confirmation by independent methods, such as FISH analyses.

### 5.2. Therapeutic Response

It is possible that measurements of clonal heterogeneity can be useful irrespective of their utility in routine diagnostics. Substantial clonal heterogeneity between primary and metastatic tumors can pose a barrier to targeted therapy. Since biopsies of metastases are frequently unavailable, it would be worth investigating if the clonal heterogeneity of primary tumors is indicative of clonal divergence in metastatic outgrowths. The index of clonal diversity is a strong predictor for malignant progression in Barrett’s esophagus [13]; thus, it can be used for guiding treatment decisions. Although experimental evidence is still lacking, it is likely that Barrett’s esophagus is not a biological oddity and that clonality measurements will turn out to be of predictive value for malignant progression in other cancers as well. However, sampling multiple regions from the same tumor is impractical for most cancer types, and it is not yet clear if obtaining accurate clonality measurements can be achieved using current routine diagnostic methods. Also, though multiple biopsies from a single tumor might be impractical, the degree of cellular and molecular heterogeneity within a relatively small sample might also turn out to be of predictive value.

Biological interactions between tumor clones could have a dramatic impact on tumor evolution, both in natural progression and in evolution in response to selection forces created by therapeutic interventions. This suggestion is supported by experimental studies in mice that have demonstrated that the growth of tumors resulting from transplanting mixtures of clones cannot be predicted from the behavior of tumors developed from individual clones [305]. Similarly, experimental tumors composed of multiple clones display different sensitivity to cytotoxic drugs compared to monoclonal tumors, as clonal interactions can either potentiate or inhibit therapeutic efficacy. Therefore, clonal heterogeneity adds an additional level of complexity to our understanding of the biology of tumor development and poses challenges for the development of successful therapies. As the field of cancer treatment is moving toward the development of targeted therapeutic approaches, the question of whether the expression of a therapeutic target in a biopsy is representative of the whole corresponding tumor becomes of primary importance [306]. For example, clonal heterogeneity involving different mutational statuses was documented for *KRAS* [301], tyrosine kinase growth factor receptors such as *EGFR* [307,308], and *TP53* [13,309] genes, for which targeted therapy is currently being developed. Heterogeneous expression of the therapeutic target does not necessarily mean that the tumor will not be responsive to the treatment, especially if the targets are responsible for the “common good”. For example, targeting an angiogenic clone can also eliminate non-angiogenic “free rider” clones, which are dependent on the secretion of angiogenic factors. Still, clonal heterogeneity needs to be accounted for in the development of targeted therapy approaches, considering that high genetic heterogeneity of tumors means high probability of pre-existent clones that are resistant to therapeutic intervention and can be selected for by therapy, resulting in therapy failure.

On the other hand, clonal heterogeneity can potentially be exploited for therapeutic benefit. Maley *et al.* proposed two such strategies of anti-cancer therapy. First, boosting the selective advantage of benign clones can be used to drive malignant clones to extinction. Second, the selective advantage of clones sensitive to a particular therapy can be boosted, allowing them to achieve clonal dominance, and then treatment can be applied. *In silico* modeling confirmed that these strategies can be much more efficient compared to standard cytotoxic therapies [310]. Unfortunately, these strategies are not yet feasible, as we lack the knowledge of how to single out or boost benign or chemosensitive clones. Gatenby and co-authors have recently proposed another strategy, termed *adaptive therapy*, which both accounts for the heterogeneity of tumor cells and takes advantage of it [219]. The conventional approach of adjuvant therapy is to hit tumors with the highest tolerated dose of cytotoxic drugs in a hope to kill the largest possible numbers of tumor cells, thereby decreasing the probability of the emergence of resistant mutants. However, this strategy would be expected to fail if resistant clones already exist before the treatment starts. Indeed, despite initial therapeutic responsiveness, treatment almost inevitably ends with the outgrowth of resistant tumors. Instead of completely eradicating tumors (which is not practically achievable in most cases), adaptive therapy aims to keep tumor size at bay by adjusting drug dose and timing of drug administration based on tumor response. This strategy prevents resistant variants from reaching fixation by maintaining the dominance of therapy-sensitive variants. Importantly, the feasibility of adaptive therapy is not contingent on knowledge of the exact mechanisms of the resistance, though the applicability of this idea has yet to be confirmed.

Elimination of MDSC is a priority for cancer patients who are candidates for active immunotherapy. Likewise, limiting the accumulation and retention of MDSC during chronic inflammation may reduce the risk of developing cancers. The proinflammatory mediators that induce MDSC are particularly attractive targets for limiting this suppressor cell population, although there are several unknowns that make it difficult to decide which mediators to target. For example, it is not known whether the various proinflammatory factors induce MDSC through independent or overlapping pathways. If the pathways are independent, then it will be necessary to block individual pathways. In contrast, if the pathways merge, then a single drug aimed at a common molecule may be effective. Future studies may identify additional proinflammatory mediators or factors that independently or coordinately regulate the accumulation of MDSC. Regardless of the complexity of MDSC induction, reduction of this inhibitory population is essential, and a comprehensive understanding of the proinflammatory mediators that regulate MDSC will provide valuable information for future drug design.

## 6. Conclusions and Future Directions

The question of whether tumors are genetically homogeneous or rather consist of a number of genetically distinct clones is not of mere intellectual curiosity. Co-existence of distinct clones within a tumor can have profound clinical implications for disease progression, diagnosis, and therapeutic responses. Recently the intratumoral heterogeneity has been the subject of attention regarding response to therapy, concluding that a better result would be obtained by targeting several pathways [307,311]. This practical aspect is essential for a more effective personalized therapy, but there are biological aspects that would be worth considering. The neoplastic transformation evolves over a period of time, involving the phenotypic progression of tumor cells along with the interaction of the initiated cell with its microenvironment. The elucidation of the steps of cancer progression relates to the acquisition of invasive capability in intraepithelial lesions and metastatic potential in invasive malignancies and is of utmost importance in the differential diagnosis of neoplasms and in the establishment of more efficacious therapeutic regimens. This functional characterization of the particular stage of tumor will certainly allow for better diagnosis, staging, prognostication and treatment of cancers.

Large amounts of evidence support the close clonal relationship of primary tumors and metastatic outgrowths. However, there is also strong evidence that suggests that metastatic spread can be an early event during tumor progression; thus, primary and metastatic tumors might become quite distinct genetically as they evolve. Some of the observed discrepancies might stem from technical issues, but it is plausible that the development of spontaneous human tumors is not confined to a single scenario. It is also possible that while many tumor cells are capable of early dissemination, only tumor cells that have acquired large numbers of oncogenic mutations are capable of initiating secondary tumors at metastatic sites.

### 6.1. Future Directions

Despite the substantial clinical significance of the issue of clonal heterogeneity, the subject remains very poorly explored. Therefore, a more systemic approach is needed to characterize the extent of clonal heterogeneity of different types and subtypes of cancers through different stages of tumor development, including disseminated tumor cells and metastases. Whether clonal heterogeneity changes in response to chemotherapy needs to be determined as well. Comprehensive characterization of tumor clonality is contingent on the development of approaches that can combine a focus on “driver” lesions with large-scale analysis. Advances in our understanding of the genetic composition of cancers make these analyses potentially feasible; however, methodology allowing for robust and cost-efficient analyses of small tumor samples is still lacking.

Accounting for the biological interactions among distinct clonal populations within tumors might pose the biggest challenge. The co-existence of multiple clones within a tumor leads to a complex network of interactions both among the clones and between the clones and the tumor microenvironment. Thus, tumors are likely to behave as complex systems and should be treated as such, especially given that more simplistic views of tumors as homogeneous entities appear to provide satisfactory results only in model systems where the complexity of experimental tumors is low. While deciphering the full extent of the interactions occurring within tumors may be an impossible task, we might be able to identify critical links and use this knowledge to control or eliminate tumors. The knowledge from the fields of evolutionary biology and ecology provides key insights for improving the clinical management of cancer patients.
